# Bayes Factor Tests for Group Differences in Ordinal and Binary Graphical Models

**DOI:** 10.1017/psy.2025.10060

**Published:** 2025-11-04

**Authors:** Maarten Marsman, Lourens Jan Waldorp, Nikola Sekulovski, Jonas Haslbeck

**Affiliations:** 1 Universiteit van Amsterdam, Netherlands; 2 Department of Clinical Psychological Science, Maastricht University, Netherlands

**Keywords:** Bayes factor, graphical model, network comparison, network psychometrics, ordinal variables

## Abstract

Multivariate analysis of psychological variables using graphical models has become a standard analysis in the psychometric literature. Most cross-sectional measures are either binary or ordinal, and the methodology for inferring the structure of networks of binary and ordinal variables is developing rapidly. In practice, however, research questions often focus on whether and how networks differ between observed groups. While Bayes factor methods for inferring network structure are well established, a similar methodology for assessing group differences in networks of binary or ordinal variables is currently lacking. In this article, we extend the Bayesian framework for the analysis of ordinal Markov random fields, a network model for binary and ordinal variables, and develop Bayes factor tests for assessing parameter differences in the networks of two independent groups. The proposed methods are implemented in the R package bgms, and we use numerical illustrations to show that the implemented methods work correctly and how well the methods work compared to existing methods in situations resembling empirical research.

## 1 Introduction

Multivariate analysis of psychological variables using graphical models has become a staple of the psychometric literature (Marsman & Rhemtulla, 2022). The two most popular graphical models for cross-sectional data in psychology are the Ising graphical model (IGM; Ising, 1925) for networks of binary variables and the Gaussian graphical model (GGM; Dempster, 1972) for networks of continuous, normally distributed variables. Although popular, these graphical models do not fit the nature of most cross-sectional psychological data, which are typically scored on a Likert-scale and thus ordinal. To fill this gap, we proposed the ordinal Markov random field in earlier work (OMRF; Marsman et al., 2025). The IGM, GGM, and OMRF are Markov random fields (MRFs; Kindermann & Snell, 1980), and their edge weights reflect the conditional dependence between pairs of variables in the network. Several approaches exist to estimate these network structures from empirical data (e.g., Borsboom et al., 2021), and the more recent Bayesian approaches have the unique advantage of being able to test whether a pair of variables in the network are conditionally independent or not (Sekulovski et al., 2024; Williams & Mulder, 2020a).

In many network studies, the central research question is whether networks differ between two or more observed groups, such as people with a diagnosis vs. people without a diagnosis. There are several methods to test for group differences in network structure (see Haslbeck, 2022, for a recent review). The network comparison test (NCT; van Borkulo et al., 2023) is a widely used permutation test for assessing parameter equivalence across groups in IGMs, GGMs, or mixed graphical models (MGMs; Haslbeck & Waldorp, 2020). The fused graphical lasso (FGL; Danaher et al., 2014) is an alternative approach that has an additional penalization parameter for group differences in GGMs, and Epskamp et al. (2022) proposed an iterative model search approach within an SEM framework. Recent work has also explored network comparisons at the level of latent dimensionality or community structure (e.g., Jamison et al., 2024). All of these methods are based on frequentist approaches and as such cannot support the null hypothesis of equivalence, only reject it. Williams et al. (2020) proposed a Bayesian approach to assessing group differences in GGMs based on the Bayes factor (Jeffreys, 1961; Kass & Raftery, 1995), which quantifies the strength of evidence for the competing hypotheses of parameter equivalence or invariance and parameter differences. An important advantage of a Bayes factor test is that it can support the null hypothesis of parameter equivalence, *the evidence for the absence of a difference*, but it can also separate this conclusion from a lack of support for either hypothesis, *an absence of evidence*. These advantages of the Bayes factor contribute to a robust methodology for network analysis (Huth et al., 2023), especially network comparisons. However, this type of analysis is not currently available for OMRFs.

Here, we address this problem by presenting an extension of the OMRF to two independent groups and developing a Bayesian methodology for assessing group differences in the estimated networks. Since the IGM is a special case of the OMRF, this also provides a test for group differences for the IGM. We develop a Bayesian methodology to assess parameter differences in the OMRF in a manner similar to how we test for mean differences in the Bayesian independent samples *t* test (Rouder et al., 2009). Central to the Bayesian *t* test is the parameterization of the model so that the group difference is a parameter and this effect is separated from the overall mean. The Bayes factor test then pits the hypothesis that the group difference parameter is zero against the alternative hypothesis that the parameter is free to vary. Here, we apply this strategy to each of the OMRF parameters.

In a recent review of Bayesian methods for testing conditional independence, Sekulovski et al. (2024) distinguished between two types of Bayes factors: the “single-model” and the “inclusion” Bayes factors. The single-model Bayes factor, typically derived via the Savage–Dickey density ratio (Dickey, 1971), requires strong assumptions about the presence or absence of other parameter differences when testing for a specific effect, and may be sensitive to violations of these assumptions (Sekulovski et al., 2024). In contrast, the inclusion Bayes factor uses Bayesian model averaging (BMA; Hinne et al., 2020; Hoeting et al., 1999) to account for model uncertainty by averaging over all possible configurations of parameter differences. This allows each effect to be tested while integrating over the uncertainty in the rest of the model. An important advantage of this approach is that it can directly account for multiplicity through the prior distribution on model space, something that the single-model Bayes factor cannot account for. We therefore base our methodology on the inclusion Bayes factor, extending its application from conditional independence testing (Marsman et al., 2025) to the problem of assessing group differences in OMRF parameters.

The remainder of this article is organized as follows. First, we provide a theoretical overview of the OMRF and its extension to account for group differences in Section 2. We also present new one- and two-group extensions for ordinal variables with a neutral response category in the Supplementary Material. Consistent with the Bayesian approach of Marsman et al. (2025), we use the pseudolikelihood (Besag, 1975) because the normalization constant of the OMRF is computationally intractable. We introduce the pseudoposterior and outline a Bayesian procedure for its estimation in Section 3. Then, in Section 4, we detail the Bayesian approach to hypothesis testing in the general case of the OMRF and, in particular, testing for group differences in model parameters. Throughout these two sections, we use numerical checks to verify that the methods work as intended. In additional simulations in Section 5, we compare the proposed approach with two existing methods for assessing group differences that must treat the ordinal data as Gaussian (i.e., use a GGM); the NCT and the single-model Bayes factor proposed by Williams et al. (2020). Finally, we discuss how our method can contribute to more robust results in the network literature and suggest avenues for future research.

## 2 Markov random field graphical models for ordinal data

### 2.1 The ordinal MRF

The OMRF was independently studied by Anderson & Vermunt (2000) and Suggala et al. (2017), and was later formalized as a graphical model for multivariate ordinal data by Marsman et al. (2025). It generalizes the well-known IGM, which arises as a special case when each variable has only two response categories. In this section, we present the OMRF as a multivariate generalization of the adjacent category logit model for ordinal responses (Agresti, 2010, 2018).

Suppose we have a univariate ordinal variable 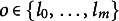 , where 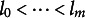 are ordered response labels, and defining the corresponding category index 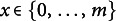 such that 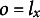 and equivalently 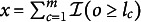 . The adjacent category model characterizes the log-odds between successive categories as 

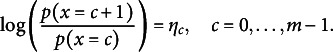

To ensure that the model parameters are identifiable, we set 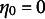 . The resulting probability mass function is given by 

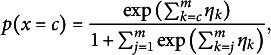

which defines a member of the exponential family with canonical parameters 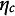 and sufficient statistics 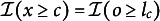 .

Suppose now that we have 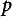 ordinal variables 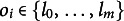 , for 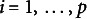 , and defining the corresponding category indices 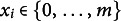 ^1^ such that 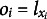 and equivalently 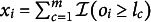 . We can regress a given variable 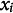 on the others by translating the canonical parameters into a linear model 

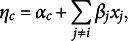

where 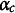 are category-specific intercepts and 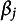 are common slopes. The corresponding adjacent category model is 

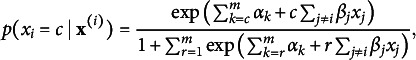

where 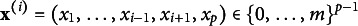 denotes the vector of all variables except 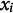 .

The formulation of the adjacent category model above depends on the ordinal variables only through the sufficient statistics 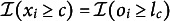 and the interaction terms 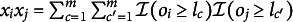 . Because these terms are computed from cumulative indicators, the model captures only the ordering of the categories, without relying on any numeric interpretation or spacing of the labels. It is therefore invariant under monotonic recodings of the ordinal variables and does not assume interval-scale measurement. Although the model represents ordinal categories using integer-valued statistics, this encoding reflects only the order, not the scale, of the responses. In contrast to this, commonly used ordinal regression models, such as the cumulative logit and continuation ratio models (Agresti, 2010), lack sufficient statistics that map ordinal responses to interpretable indices. Moreover, as shown by Suggala et al. (2017), these models do not correspond to full-conditionals of a joint distribution, and are therefore not compatible with a multivariate MRF formulation.

The adjacent category model, on the other hand, does yield consistent full-conditionals and corresponds to the following joint probability model over 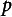 ordinal variables (Suggala et al., 2017): (1)

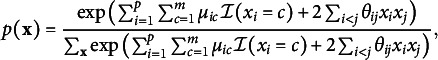

where 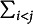 denotes 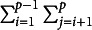 , and the denominator sums over all possible realizations of 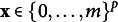 . In this formulation, the category threshold parameters 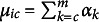 for variable 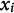 are derived from the intercepts in the adjacent category model. They represent the relative tendency to respond in category 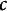 over the baseline category 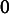 (because we fixed 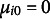 ), with larger values indicating a greater tendency of responding in category 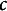 . Comparisons between 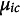 and 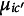 reflect relative preference among non-baseline categories. The interaction parameters 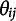 are symmetric and correspond to the regression weights, with 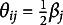 from the model regressing 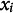 on 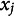 , or equivalently, 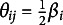 from the model regressing 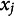 on 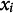 . These parameters capture the strength of the *partial association* between variables 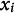 and 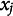 , reflecting the conditional dependence that remains after accounting for all other variables in the network.

### 2.2 Extending ordinal MRFs to two independent samples

We now consider the scenario where we have ordinal data from two groups. The goal of this article is to assess if and how the networks of ordinal variables differ between the two groups. The OMRF is characterized by two types of parameters, the category thresholds 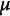 and the pairwise interactions 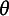 , and thus we want to assess the differences in terms of these two parameters. In order to assess whether there are differences in these two parameters between the two groups in a Bayesian framework, it is ideal if we can explicitly parameterize these differences. In this section, we formulate these parameterizations.

We model the differences in category thresholds between the two groups by reformulating the category thresholds as follows: (2)

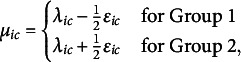

where 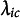 is the overall category threshold, i.e., the category threshold we would find if the data from the two groups were combined and analyzed as a single data set. The parameter 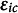 is then the threshold difference effect, i.e., the category threshold for Group 2 minus that for Group 1. If it is zero, there is no difference in the category thresholds between the groups, and the category threshold parameter in each group is equal to the overall threshold parameter 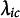 . However, if the threshold difference effect is not zero, there is a difference in the category thresholds between the two groups.

We model the difference in the pairwise interaction between the two groups in a similar way, reformulating the pairwise interactions as follows: (3)

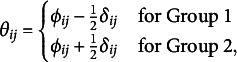

where 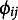 is the overall pairwise interaction, i.e., the pairwise interaction we would find if the data from the two groups were combined and analyzed as a single data set. The parameter 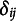 is then the pairwise difference effect. If it is zero, there is no difference in the pairwise interactions between the groups, and the pairwise interaction in each group is equal to the overall pairwise interaction 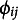 . However, if the pairwise difference effect is not zero, there is a difference in the pairwise interactions between the two groups.

**Figure 1:**
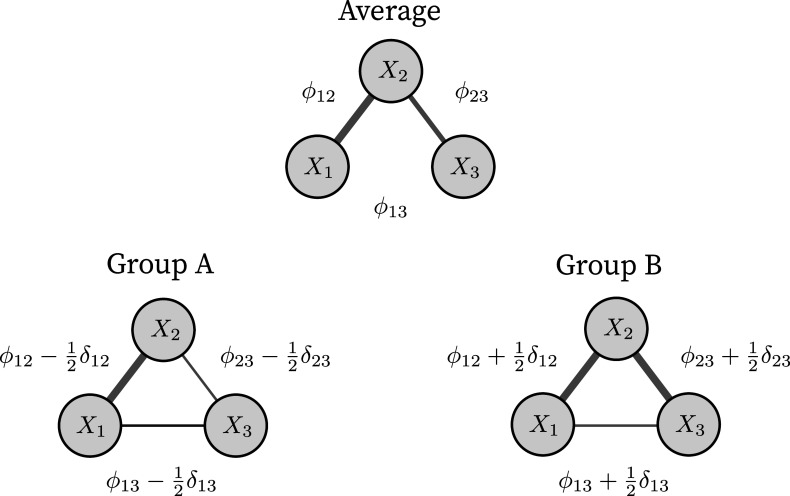
This illustrates how the pairwise interactions and their differences are modeled for the two groups. *Note*: The top panel shows the average size of the pairwise interactions, 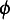 , that would be obtained if grouping were ignored. The bottom two panels show the pairwise interactions for the two groups. Note that there is no group difference in the interaction between variables one and two ( 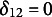 ), and the group parameters are equal to the average, 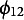 . In contrast, the interaction between variables one and three switches sign and is here fully dictated by the difference effect, 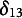 . Finally, the interaction effect between variables two and three differs in size. Group A has a smaller interaction effect, and group B has a larger interaction effect.

Note that the choice of the factor 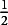 in Eqs. (2) and (3) serves to evenly distribute the group difference parameter across both groups. This parameterization defines 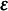 and 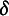 as contrasts between Group 2 and Group 1 (e.g., 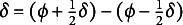 ), such that their sum or average equals zero. As a result, the shared or overall effects 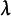 and 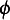 are defined as the average across the two groups (e.g., 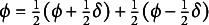 ). While alternative parameterizations are possible, such as those using projection matrices to handle more than two groups (e.g., Rouder et al., 2012), our focus here is on the two-group case. This provides a natural and interpretable foundation that can be extended in future work to more general group structures. Figure 1 illustrates the approach to modeling the pairwise interactions of a three-variable network in two groups.

We now express the OMRF separately for each group, using the parameterizations in Eqs. (2) and (3). Let 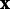 denote the response vector for a participant in Group 1, and 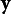 for a participant in Group 2. The joint distribution of the ordinal variables in each group is then given by (4)

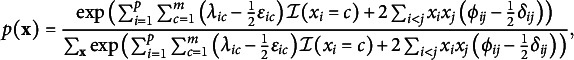


(5)

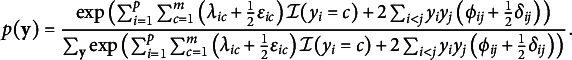

These group-specific formulations encode between-group differences explicitly through the parameters 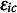 (for category thresholds) and 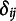 (for pairwise interactions). When both are zero, the models reduce to a shared structure defined by the common parameters 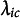 and 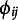 . We prove the identifiability of the two-group OMRF in Appendix A.2.

## 3 Estimation

We adopt a Bayesian approach to estimate the parameters of the two-group OMRF introduced in Section 2. This extends the estimation framework proposed by Marsman et al. (2025) for a single group to the case of two independent samples.

### 3.1 Data and likelihood

Let 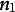 and 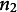 denote the number of individuals in Group 1 and Group 2, respectively. Let 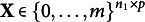 and 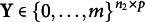 be the observed response matrices, where each row corresponds to a response vector 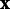 or 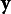 from an individual in the respective group. We assume that individuals are independent within and between groups. Under these assumptions, the likelihood of the observed data is given by (6)

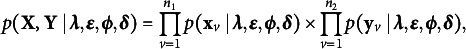

where 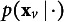 and 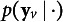 are the group-specific models defined in Eqs. (4) and (5).

#### 3.1.1 Likelihood intractability and the pseudolikelihood approximation

The likelihood in Eq. (6) is computationally intractable because it involves normalization constants that are prohibitively expensive to compute. Each group-specific model (Eqs. (4) and (5)) requires summing over all possible configurations of the *p* ordinal variables, each taking 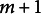 possible values. As a result, the normalization constant for each group involves 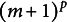 terms. For example, with just 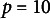 variables rated on a 5-point Likert scale ( 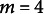 ), each normalization constant requires summing over 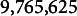 combinations. Since the normalization constants must be evaluated repeatedly during MCMC sampling, this renders full-likelihood-based inference computationally infeasible for even modestly sized networks.

While several methods have been proposed to circumvent the need to compute normalization constants in models with intractable likelihoods (for a review, see Park & Haran, 2018), the pseudolikelihood approach introduced by Besag (1975) remains the most widely used strategy in network psychometrics. It is a special case of the broader class of composite likelihood methods, which combine low-dimensional marginal or conditional components to approximate the full likelihood (Varin et al., 2011). Its computational efficiency and strong empirical performance in structure selection have also contributed to its growing popularity in Bayesian modeling (e.g., Marsman et al., 2025; Mohammadi et al., 2023; Vogels et al., in press). The pseudolikelihood replaces the full multivariate distribution, such as the distribution of the ordinal response vector 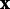 in Eq. (4), with the product of its full conditionals: 

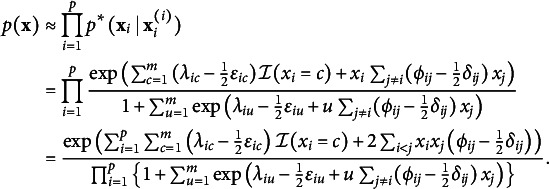

This approximation preserves the structure of the full model but replaces its intractable normalization constant 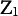 with a product of tractable, one-dimensional normalization factors. For example, in the ten-variable network example with five ordinal categories ( 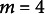 ), the full normalization constant contains 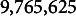 terms, while the pseudolikelihood normalization contains only 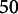 terms. This dramatic reduction in computation makes the pseudolikelihood particularly attractive for inference in high-dimensional settings.

Although the pseudolikelihood is a relatively crude approximation to the full likelihood, the generalized posterior it induces remains consistent (Miller, 2021). For both the IGM and the OMRF, it has been shown that the pseudolikelihood introduces no additional bias beyond that already present in the full likelihood (Keetelaar et al., 2024; Marsman et al., 2025). Moreover, multiple studies have demonstrated that pseudolikelihood-based graph recovery is consistent in high-dimensional settings, where both the number of variables and observations grow (e.g., Barber & Drton, 2015; Ravikumar et al., 2010). These results also extend to Bayesian settings (Csiszár & Talata, 2006; Pensar et al., 2017). However, despite its favorable theoretical properties, the pseudolikelihood is known to underestimate standard errors (e.g., Keetelaar et al., 2024), and consequently, posterior variances. As a result, edge selection procedures may become overly sensitive to small, spurious effects.

Replacing the full likelihood in Eq. (6) with the pseudolikelihood yields a *pseudoposterior distribution*, which we use for inference: 

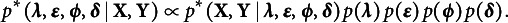

Here, 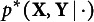 denotes the pseudolikelihood defined by the product of full conditionals for each group. Although this is not a proper posterior in the strict Bayesian sense, theoretical results show that the pseudoposterior remains consistent under mild conditions (Miller, 2021; Pauli et al., 2011; Ribatet et al., 2012). Bayesian inference under the pseudoposterior requires specifying prior distributions for the model parameters, which we outline in the next section.

### 3.2 Prior specification

The two-group OMRF has two types of parameters. The category threshold 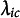 and the pairwise interaction parameters 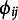 that would be obtained if the data sets from the two groups were combined. If the only goal of the analysis is to test group differences, these parameters are nuisance, and only the threshold difference parameters 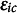 and the pairwise difference parameters 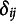 are of interest.

We follow Marsman et al. (2025) for the specification of prior distributions for the nuisance parameters. Independent beta-prime distributions are specified on exponentially transformed category threshold parameters 
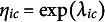
 , or equivalent, 

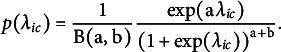



In line with the prior setup of Marsman et al. (2025), independent Cauchy distributions with a scale of 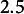 are specified for the pairwise interaction parameters.

We also use independent Cauchy distributions for the two sets of difference parameters. This choice lays the foundation for our testing procedure, which we describe later, and which includes a variable selection procedure based on independent spike and slab prior distributions. The Cauchy distribution is a popular candidate for testing because of its tail behavior. We will use a scale of 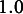 .

### 3.3 A Gibbs sampling procedure for the pseudoposterior distribution

The full Bayesian framework is approximated by combining the pseudolikelihood with the prior distributions specified in the previous section, yielding a pseudoposterior distribution over the model parameters. As this distribution is not available in closed form, we use numerical methods to approximate it.

Specifically, we employ a Gibbs sampling algorithm (Geman & Geman, 1984) to draw dependent samples from the joint pseudoposterior of the two-group OMRF. At each iteration, the algorithm cycles through updates of the four parameter blocks— 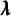 , 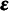 , 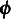 , and 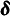 —by drawing from their full conditional pseudoposterior distributions. Since these conditional distributions are not of a known form, we use Metropolis–Hastings (Tierney, 1994) algorithms to simulate from them. The general procedure is summarized in Algorithm 1, with full technical details provided in Appendix B.

**Figure:**
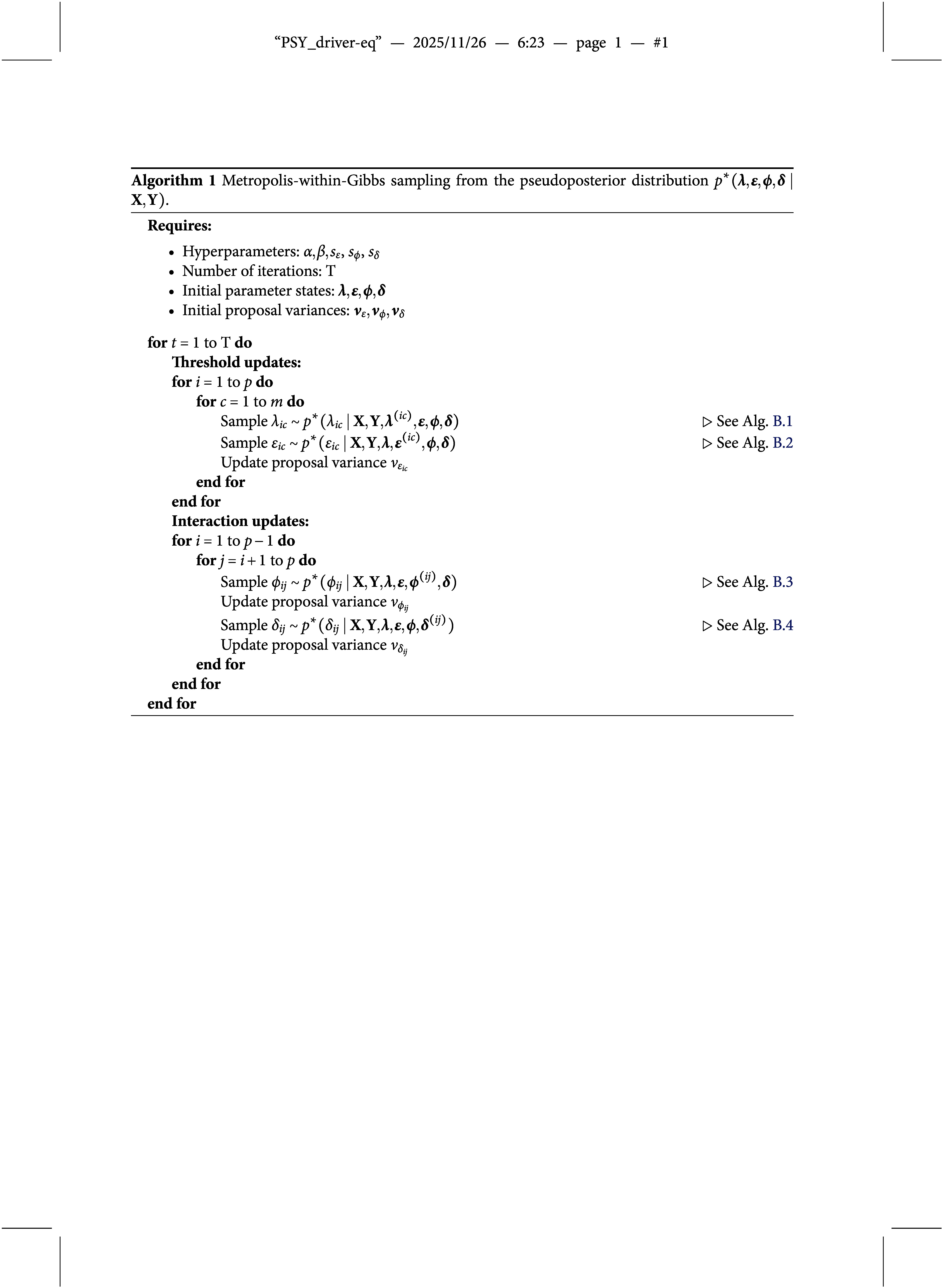

This algorithm is implemented in the bgmCompare() function of version 0.1.4.2 of the bgms R package (Marsman et al., 2024), and serves as the computational backbone of our hypothesis testing framework. In bgms, the Gibbs sampler runs in two stages. The burn-in phase (default: 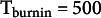 iterations) initializes parameters at zero, tunes proposal variances (initially set to one), and guides the chain toward regions of high posterior density. This is followed by the main sampling phase (default: 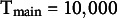 iterations), during which the samples are collected for inference. Both parameter values and proposal variances are carried over from the burn-in stage.

Numerical Check I in Appendix C.1 shows that the MwG procedure is correctly implemented in bgms, and that its posterior estimates agree with those from an alternative rstan implementation.

## 4 Hypothesis testing

### 4.1 Bayes factor tests for conditional independence in ordinal MRFs

A key advantage of the OMRF is that hypotheses about the conditional dependence and independence of ordinal variables *i* and *j* can be tested via their partial association 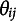 . Specifically, 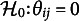 indicates conditional independence, and 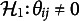 indicates conditional dependence. We use the Bayes factor (Jeffreys, 1961; Kass & Raftery, 1995), which is defined as the change in beliefs about the relative plausibility of the two hypotheses before and after observing the data: (7)

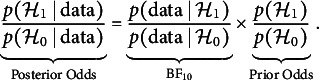

The term on the left, the posterior odds ratio, conveys the relative plausibility of the two competing hypotheses after viewing the data. The formula above shows that the posterior odds can be expressed as the product of two factors: The Bayes factor, 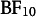 , which indicates the relative support for the two hypotheses in the data at hand, and the prior odds, the relative plausibility of the two hypotheses before having seen the data.

The subscripts in the Bayes factor notation indicate the direction in which the support is expressed. 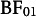 indicates the relative support for 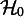 over 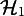 , and 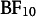 indicates the relative support for 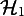 over 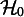 : Note that the Bayes factor 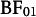 is the reciprocal of 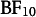 , i.e., 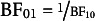. Values greater than 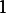 indicate that there is relative support for 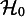 in the data, while values less than 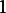 indicate relative support for 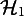 . When the Bayes factor is 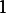 , both hypotheses predict the data equally well.

#### 4.1.1 Bayes factor test for conditional dependence

Marsman et al. (2025) introduced binary indicator variables 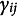 (as commonly used in Bayesian variable selection; see, e.g., Dellaportas et al., 2002; George & McCulloch, 1993; Kuo & Mallick, 1998) to explicitly model the presence or absence of an association between variables *i* and *j*, and used them to define the following spike-and-slab prior on the partial association 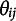 : 

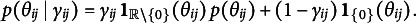

Here, 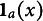 is an indicator function that restricts 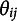 to the appropriate region of the parameter space, conditional on the value of 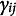 (this formulation is due to Gottardo & Raftery, 2008). When 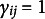 , the association is considered present, and 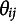 is treated as a free parameter with a continuous prior density (e.g., a Cauchy distribution). In contrast, when 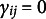 , the association is absent, and the parameter is fixed to zero.

Because the indicator variable 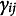 directly encodes whether a partial association 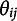 is present or absent, hypotheses about conditional dependence between variables *i* and *j* can be naturally expressed in terms of 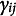 : 

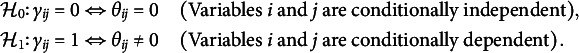

In this formulation, testing for conditional independence reduces to testing whether 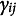 equals zero.

From Eq. (7), we see that the Bayes factor can be defined as the ratio of the posterior odds to the prior odds of the competing hypotheses: 

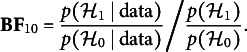

In the current context, where the hypotheses are defined in terms of the indicator variable 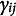 , the Bayes factor becomes: 

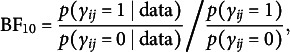

i.e., the posterior odds of including the association divided the prior odds.

Marsman et al. (2025) proposed the use of Bernoulli 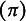 and Beta 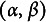 -Bernoulli priors on the indicator variables, and developed an MCMC algorithm to estimate the joint posterior distribution of OMRF parameters and indicator variables. This framework provides the basis for computing the inclusion Bayes factor and has recently been extended with a stochastic block prior on the indicator variables to model network clustering (Sekulovski et al., 2025). Note that when using the Bernoulli 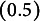 or the Beta 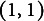 -Bernoulli prior, the prior odds are equal to one, and the Bayes factor simplifies to the posterior odds of edge inclusion.

The posterior inclusion probability for the association between variables *i* and *j* corresponds to the (marginal) posterior expectation of the indicator variable 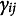 : 

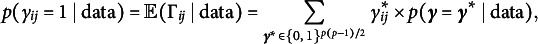

where the sum is over all 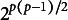 possible network structures, i.e., configurations of the vector of indicator variables 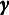 .

Since the exact sum is computationally infeasible except for small networks, we estimate it using posterior samples. Let 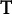 denote the number of post-burnin MCMC samples, and let 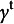 , for 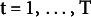 , be the sampled values of 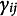 . The posterior inclusion probability is then approximated by the Monte Carlo estimate: 

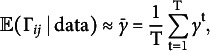

that is, the proportion of posterior samples in which 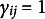 .

### 4.2 Bayes factor tests for group differences in ordinal MRFs

The Bayes factor approach to testing for conditional independence carries over seamlessly to testing for differences in the parameters between two groups. We first consider Bayes factor tests for differences in the category threshold parameters, and then for differences in the pairwise interactions.

#### 4.2.1 Group differences in category threshold parameters

We assess group differences in category threshold parameters by testing, for each variable in the network, whether the entire vector of group differences is zero. Formally, the null hypothesis states that all category thresholds for a given variable are invariant across groups— 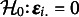 —while the alternative allows for differences in one or more thresholds— 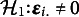 . Testing the full vector jointly reduces the number of comparisons and reflects that changes in one threshold typically affect others. That is, due to their cumulative interpretation, a difference in one threshold typically implies a shift in the overall response distribution, making it likely that one or more of the remaining thresholds for that variable will also differ between groups.

Because threshold parameters correspond to specific response categories, their estimation requires that each category be observed in both groups. If a category is present in one group but entirely absent in the other, the corresponding threshold—and hence its group difference—cannot be identified. To address this, bgms offers two modeling strategies. The first strategy is to estimate the thresholds independently in each group without testing for differences. This approach is selected by setting the main_difference_model option to “Free.” In this case, unobserved categories in one group are collapsed, but the other group’s thresholds remain unaffected. For example, if variable *i* has categories 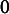 , 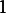 , and 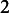 , but category 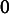 is unobserved in Group 1, then Group 1’s responses are recoded as 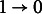 and 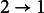 , resulting in one threshold estimate for Group 1 and two for Group 2. The second strategy is to ensure comparability by collapsing categories symmetrically across groups when a category is unobserved in either one. This is selected using the main_difference_model option set to “Collapse.” In this case, the unobserved category is collapsed into the next lower category (or the next higher one if the lowest category is missing). If a category is unobserved in both groups, it is automatically collapsed.

Building on the framework used for testing conditional dependence, we introduce binary indicator variables to explicitly model the presence or absence of group differences in the category threshold parameters. Specifically, for each variable *i*, we define a vector of group difference parameters 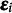 and use an indicator 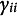 to define the spike-and-slab prior on the vector of threshold differences 

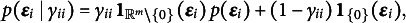

where *m* is the number of category thresholds for variable *i*. The indicator function 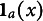 again ensures that 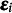 lies in the appropriate region of the parameter space, conditional on the value of 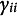 . When 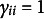 , at least one threshold differs between groups, and the differences are treated as free parameters with continuous prior densities (e.g., a Cauchy distribution). In contrast, when 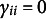 , the thresholds are invariant, and the difference vector is fixed to zero. Accordingly, our hypotheses about group differences or equivalence can be formulated in terms of 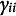 : 

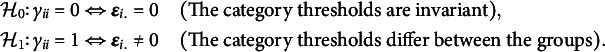

Testing for 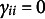 is thus equivalent to testing for parameter invariance of the category thresholds for variable *i*.

The inclusion Bayes factor quantifies how the data update our beliefs about the presence of a group difference in the category thresholds. Expressed in terms of the difference indicator 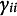 , it takes the form: 

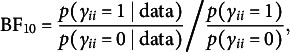

that is, the ratio of posterior to prior odds for including a difference in the thresholds for variable *i*. The corresponding posterior inclusion probabilities can be estimated from the Gibbs sampler described in Algorithm 2. A detailed discussion of the prior specification for inclusion indicators is provided in the prior specification paragraph below.

#### 4.2.2 Group differences in pairwise interaction parameters

We test for group differences in the pairwise interaction parameters. For each pair of variables 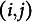 , the null hypothesis assumes no difference in the partial association between the groups— 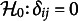 —while the alternative hypothesis suggests that there is a difference— 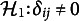 .

In line with the approach used for testing for group differences in the category thresholds, we introduce 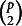 binary indicator variables 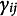 , each corresponding to a specific pairwise interaction, to use them to model the presence or absence of a group difference in the association between variables *i* and *j*, and use them to define the following spike-and-slab prior on the difference parameter 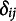 : 

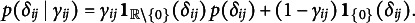

Here, 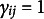 indicates that there is a non-zero difference between groups and assigns 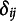 a continuous prior (e.g., a Cauchy distribution), while 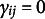 fixes the difference to zero, reflecting invariance. This formulation enables us to cast our hypotheses about group differences or equivalence in terms of the inclusion indicators: 

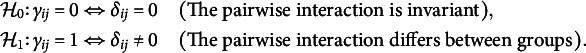

Expressed in terms of the difference indicator 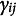 , the inclusion Bayes factor for testing whether there is a group difference in the interaction between variables *i* and *j* is given by 

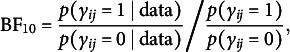

that is, the ratio of posterior to prior odds of including a difference in the pairwise interaction. The corresponding posterior inclusion probabilities can be estimated from the Gibbs sampler described in Algorithm 2.

*Prior specification for difference indicators* In bgms, prior distributions for inclusion indicators can be specified independently for category threshold differences ( 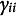 ) and pairwise interaction differences ( 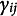 ). This separation allows researchers to express different prior beliefs about group differences in marginal distributions (thresholds) versus conditional associations (interactions). The specification of such priors and their influence on model-based inference has been studied in recent work on sensitivity analysis for Bayesian graphical models (Sekulovski et al., 2024). By default, bgms uses Bernoulli priors with fixed inclusion probabilities: 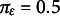 for threshold differences and 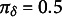 for interaction differences. These default settings imply that, *a priori*, each possible configuration of difference indicators is equally likely. But they also project group differences in category thresholds for about half of the variables and for about half of the pairwise associations, regardless of the number of effects under consideration. That is, the Bernoulli prior does not provide a correction for multiplicity (Scott & Berger, 2010). To address this, bgms also provides hierarchical priors for inclusion probabilities. Specifically, assigning a beta hyperprior, such as 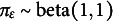 and 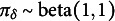 , yields a beta-Bernoulli model that induces a uniform distribution on the number of included differences. This hierarchical formulation adjusts the prior inclusion probabilities based on the total number of selected differences (e.g., Consonni et al., 2018; Marsman et al., 2022), and has been shown to provide automatic multiplicity correction (Scott & Berger, 2010).

While bgms also supports the stochastic block prior for modeling clustering in the network structure (Sekulovski et al., 2025), this functionality is not currently extended to testing for group differences. Although stochastic block priors are well suited for capturing latent community structure in psychometric networks, we do not currently consider such structured assumptions appropriate for modeling group differences in parameters. In the context of threshold and interaction differences, we do not believe that these effects follow a clustered pattern.

### 4.3 A Gibbs sampling procedure to simulate the posterior distribution

The Bayes factor tests described in the previous sections rely on the posterior distributions of the difference inclusion indicators 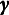 . Because these posterior distributions are not available in closed form, we estimate them using a Gibbs sampler that has the following multivariate posterior distribution as its invariant distribution: 

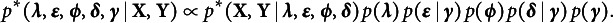

This posterior distribution extends the model from Section 3 by incorporating a hierarchical prior for the group difference parameters. Specifically, it introduces spike-and-slab priors on the group difference parameters 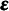 and 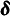 , conditional on the inclusion indicators 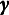 . In addition, a marginal prior is placed on the indicators themselves.

Estimating the joint pseudoposterior distribution of the model parameters and the difference indicators is a non-trivial task. Since the inclusion or exclusion of parameters changes the dimensionality of the model, a standard Gibbs sampler is not applicable. To address this, we employ a pairwise Metropolis step that jointly updates each indicator and its associated parameter (cf. Gottardo & Raftery, 2008; Marsman et al., 2025), avoiding the need for more complex reversible jump methods (e.g., Green, 1995). Specifically, we extend Algorithm 1 by incorporating a pairwise Metropolis update: for each group difference effect, we jointly update the inclusion indicator—either 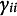 for category threshold differences or 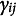 for pairwise interaction differences—along with the corresponding parameter vector 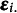 or parameter value 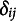 . This extension yields a valid sampling procedure for the joint pseudoposterior distribution of model parameters and difference indicators, as schematically summarized in Algorithm 2.

In the bgms R package, we run Algorithm 2 in two stages, for a total of 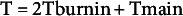 iterations. The first stage is called the burn-in and is split into two parts, each running for 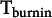 iterations. In the first part, the algorithm searches for a region of high posterior mass and calibrates the proposal variances, but does not yet update the indicator variables (as in Algorithm 1). In the second part, updates to the indicator variables are included. By default, the number of iterations in the burn-in stage in bgms is 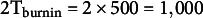 . The parameter values are initialized at zero, and the proposal variances at one. The second stage is the main simulation, during which the algorithm output is stored for further analysis.

Numerical Check II in Appendix C.2 shows that the proposed algorithm correctly recovers the prior distribution of the pairwise difference indicators and parameters, and Numerical Check III in Appendix C.3 shows that the inclusion Bayes factors based on the output of Algorithm 2 are consistent with theoretical expectations.

## 5 Numerical experiments

The goal of the numerical checks in Appendix C was to demonstrate that the proposed method yields consistent parameter estimates and selection of group differences. Here, we provide an additional numerical experiment to evaluate the performance of the method in selecting group differences in a setting similar to an empirical psychological study, and how it compares to other popular methods for comparing pairwise association networks. The R script for this numerical experiment can be found at https://osf.io/txhbp/overview?view_only=0a2ed24ecc4448fbba104faa28e6f8c7.

### 5.1 Simulation setup

The goal of this simulation study is to evaluate how well our method compares to existing methods for testing group differences in scenarios that resemble typical empirical research studies in the field of psychology.

**Figure:**
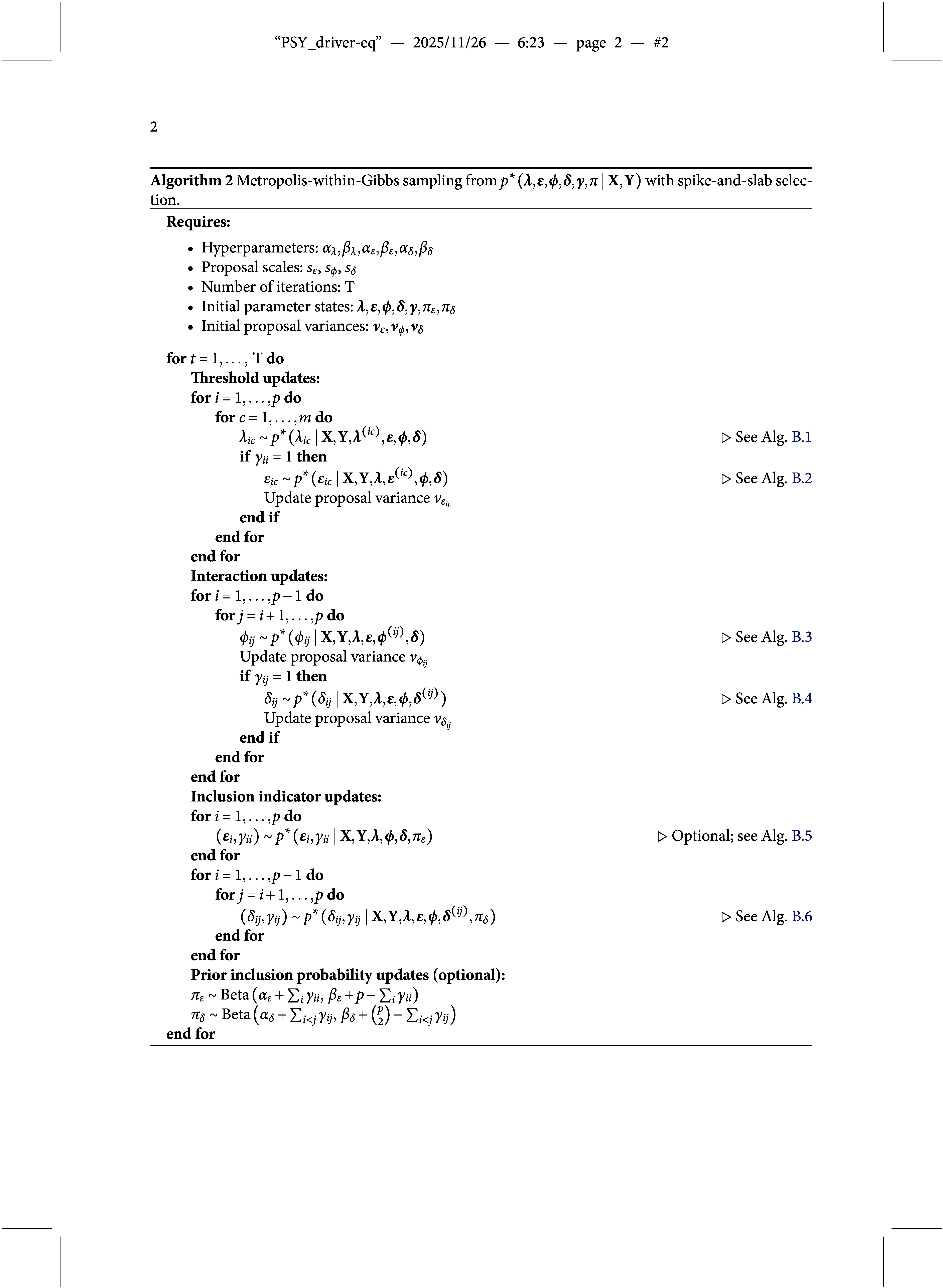

#### 5.1.1 Simulation design

In each simulation, we generate data from two groups whose distributions differ in a controlled number of interaction parameters. We systematically vary four key aspects of the data-generating process: i) the number of variables ( 
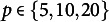
 ), ii) the population network density 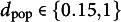 , defined as the proportion of the overall interaction parameters 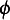 (see Eq. (3)) that are present, iii) the proportion of interaction parameters that differ between groups ( 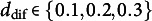 ), and iv) the sample size per group ( 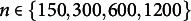 ). This results in a 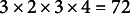 condition design, which we replicate 100 times to account for variability across repetitions.

We chose the variation in the number of variables based on the number of variables that are typically included in empirical applications of network models. The manipulation of 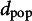 is not of primary interest in the present study, but we include it to examine whether it affects the performance of the NCT that we analyze below, which uses a permutation test based on regularized regression models that assume sparsity. The range of 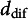 values was selected to reflect what we consider reasonable for the size and prevalence of group differences in cross-sectional psychological data.

#### 5.1.2 Data-generating model

The two-group model, defined by Eqs. (4) and (5), is characterized by two sets of parameters. The first set consists of category thresholds 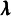 and pairwise interactions 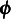 , which together characterize the overall model obtained when group membership is ignored. The second set consists of category threshold differences 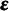 and pairwise interaction differences 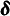 . Because the methods we compare to below assess only interaction differences, we assume throughout that 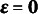 and focus on selecting 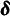 . For our simulations, we therefore need to define the overall parameters ( 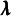 and 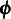 ), as well as the difference parameters ( 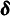 ). These choices are described in the ensuing sections.

*Selecting overall parameter values* To ensure that the overall parameters reflect realistic values, we calibrate them using empirical data. Specifically, we estimate an ordinal MRF on data from Billieux et al. (2021), which includes responses from 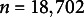 individuals to the 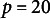 items of the short version of the UPPS-P Impulsive Behavior Scale (Cyders et al., 2014). The items include statements such as “I usually think carefully before doing anything” and are rated on a 4-point Likert scale ranging from “Agree strongly” to “Disagree strongly.” These data are representative of the ordinal response formats and content domains commonly used in psychological research, and the large sample size provides stable population-level estimates of interaction parameters. The resulting estimates for the pairwise interactions form a right-skewed distribution with a range of 
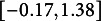
 , a mean of 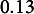 , and a standard deviation of 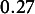 . When generating the overall model in the simulation, we first determine which interaction parameters are nonzero, and then sample values for the nonzero interactions from this empirical distribution.

Population network density is manipulated by varying the number of pairwise interactions retained in the model. For the condition 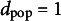 , we use the full set of pairwise interactions and corresponding thresholds estimated from the empirical data. For the condition 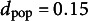 , each potential interaction is retained independently with probability 0.15. This sparsification affects the marginal distributions, because both interaction parameters and thresholds jointly determine the observed response patterns. For example, if most interactions are positive, setting many of them to zero while keeping thresholds fixed will reduce the frequency of responses in the higher categories relative to the empirical marginals. To prevent such confounding, we re-estimate the ordinal MRF on the same empirical data for each sampled sparse graph, using the selected structure as a constraint. This ensures that marginal distributions remain comparable across conditions and prevents confounding between structural sparsity and a shift in the marginal distribution. A side effect of this procedure is that the retained interaction parameters under 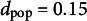 tend to be stronger than those under 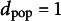 , since weak or absent edges are excluded. Although we do not have a formal account of how this shift influences the detection of group differences, lower variance in a variable is likely to reduce sensitivity to changes involving that variable. The re-estimation procedure described above mitigates this issue by aligning marginal distributions across density conditions.

*Selecting difference values* We now specify the group differences used in the simulations. While the pairwise interactions and category thresholds of the overall model are calibrated using empirical data, there is no corresponding population-level information available for group differences. In particular, the distribution of group differences depends on the grouping variable, and unlike the overall associations, it is not shaped by constraints such as questionnaire design. For instance, in empirical data, extremely skewed marginals or overly strong associations are often avoided by construction, whereas this is not the case for latent group differences.

To generate group-specific models, we follow Eq. (3) and construct the two group models defined in Eqs. (4) and (5) by subtracting or adding half of the group difference parameter from the overall interaction parameter. This ensures that the group models differ in a symmetric and centered way, while preserving the structure of the overall model.

In the absence of empirical constraints, a natural choice might be to draw the group differences from a Gaussian distribution centered at zero. However, such a distribution would result in a large number of very small differences, many of which would be of little substantive interest. Instead, we aim to simulate group differences that are meaningfully different in magnitude. While any such definition is necessarily arbitrary, we set a lower bound of 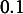 and cap the largest differences at 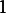 . To operationalize this, we sample values from a mixture of uniform distributions 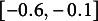 and 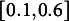 , with equal probability of positive and negative differences.

For each simulation condition, we fix a proportion 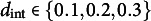 of the pairwise interactions to differ between groups. We then randomly select that proportion of interactions from the full set, and assign each a difference value (i.e., 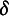 ) drawn from the mixture of uniform distributions described above. This procedure implies that the true data-generating model differs across repetitions of the simulation design, as new difference values are drawn each time. Within a single repetition, however, the same pair of group models is used to generate all data.

*Simulating the data* We sample 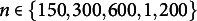 independent observations from each of the two groups. To avoid extreme marginal distributions that would make the detection of group differences difficult or lead to errors due to insufficient variance, we impose the following constraint on the generated data: we resample the entire process, both the generation of the true model and the sampling of observations, until the second-most frequent response category contains at least 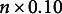 observations. Across all conditions, this constraint resulted in an average of 0.02 and a maximum of three resampling iterations.

#### 5.1.3 Selecting group differences

To evaluate the performance of our method in identifying group differences in interaction parameters, we compare the two prior formulations for the difference indicators introduced in Section 4.2.2. The first uses a Bernoulli prior with fixed inclusion probability 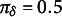 for each interaction difference. The second uses a beta-Bernoulli prior, where 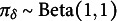 . All other prior distributions are defined as in Section 3.2, and correspond to the default values used in the bgms package. Posterior distributions are approximated using 10,000 MCMC iterations, as implemented in the R package bgms (version 0.1.4.2; Marsman et al., 2024).

We compare these two versions of our method to two widely used approaches for detecting group differences in GGMs (e.g., Haslbeck, 2022). Although these methods assume continuous data, they are often applied to ordinal data in empirical studies, where ordinal responses are treated as continuous to estimate partial association networks. The first method is a frequentist permutation test of edge equality across groups, implemented in the R package NetworkComparisonTest (version 2.2.2; van Borkulo et al., 2023). We use 10,000 permutations to generate null distributions for each edge-specific test. The second is a Bayesian approach that uses a single-model Bayes factor, implemented in the R package BGGM (version 2.1.3; Williams & Mulder, 2020b). This method compares two models: both estimate all pairwise differences except one, which is constrained to equality across groups (Williams et al., 2020). Posterior estimation is also based on 10,000 MCMC iterations.

In total, we compare four methods for selecting group differences in interaction parameters: two variants of our method and two GGM-based alternatives.

#### 5.1.4 Evaluating performance

Performance metrics, such as sensitivity, specificity, and precision, depend on the choice of a specific threshold or tuning parameter (e.g., a cutoff on inclusion probability, Bayes factor, or *p*-value). While these metrics are intuitive and often reported, they are threshold-dependent and can therefore obscure how a method performs across its full range of decision rules. This is particularly relevant in the present context, where the goal is to compare methods with different inferential philosophies and calibration strategies.

To evaluate performance independent of a specific threshold, we use the receiver operating characteristic (ROC) curve. The ROC curve plots the false positive rate (FPR, or 1—specificity; *x*-axis) against the true positive rate (TPR, or sensitivity; *y*-axis) across a range of threshold values (Fawcett, 2006). Thus, each point on the ROC curve corresponds to a specific sensitivity–specificity tradeoff. To summarize overall performance, we compute the area under the ROC curve (AUC), which ranges from 0 to 1. An AUC of 0.5 indicates performance no better than random guessing, while higher AUC values indicate better separation between true and false positives. By aggregating over the full range of thresholds, the AUC avoids the arbitrariness associated with selecting a single threshold and allows for more robust performance comparisons across methods.

### 5.2 Simulation results

**Figure 2:**
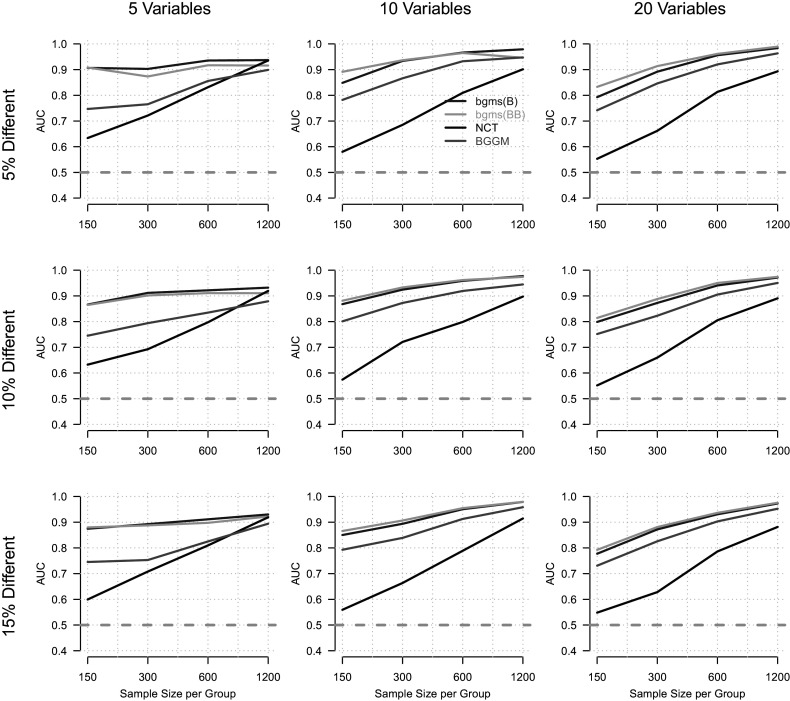
Area under the ROC curve (AUC; *y*-axis) as a function of sample size per group (*x*-axis), for different numbers of variables (columns) and proportions of true group differences (rows). *Note:* The overall model has a density of 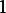 , corresponding to a fully connected network.

We first report the results for the 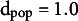 condition, in which the overall network is fully connected, in Figure 2. The figure shows the AUC as a function of sample size (*x*-axis), with separate columns for the number of variables and rows for the proportion 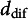 of edges that differ between groups. The four compared methods are indicated by color. As expected, the AUC increases with sample size for all methods. The few exceptions to this trend occur in conditions with fewer variables ( 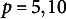 ) and are likely due to sampling variability.

When comparing the four methods, we find that the two variants of the proposed method perform best overall (mean AUC: bgms(BB) = 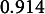 , bgms(B) = 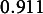 ), followed by BGGM ( 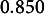 ) and the NCT ( 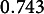 ). However, as the sample size increases, performance differences between methods diminish. In particular, the NCT closes the gap with other methods in the condition with 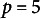 and large *n*.

**Figure 3:**
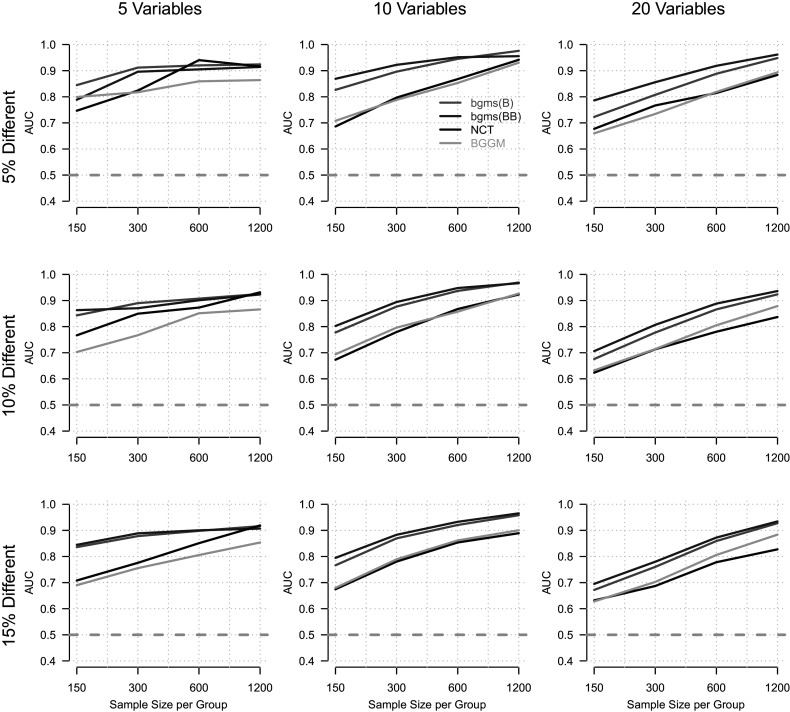
Area under the ROC curve (AUC; *y*-axis) as a function of sample size per group (*x*-axis), for different numbers of variables (columns) and proportions of true group differences (rows). *Note:* The overall model has a fixed network density of 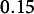 .

Figure 3 presents results for the 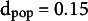 condition in which the overall network is sparsely connected. This condition was included to examine how graph sparsity affects the detection of group differences, especially given that the NCT relies on LASSO-based estimation, which assumes a sparse structure. As in the previous condition, performance improves with increasing sample size. Average performance across all methods and conditions is somewhat lower in this setting ( 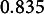 vs. 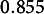 in the dense condition). The proposed methods again perform best, with bgms(BB) achieving the highest mean AUC ( 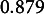 ), followed by bgms(B) ( 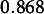 ). In this condition, NCT slightly outperforms BGGM (NCT = 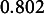 , BGGM = 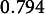 ), and again approaches the performance of the other methods at high sample sizes for 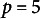 .

**Figure 4:**
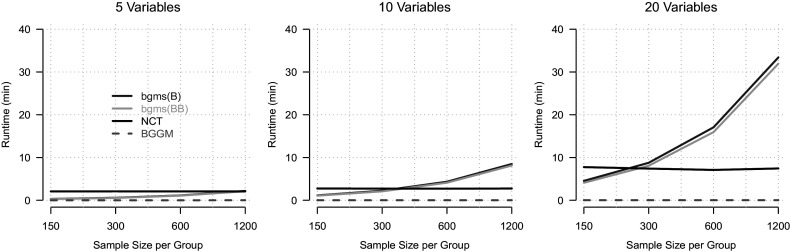
Average runtime in minutes for the four compared methods (colors) as a function of sample size per group (*x*-axis) and number of variables (columns), averaged over all other simulation conditions.

Figure 4 summarizes runtime as a function of the number of variables (columns) and sample size per group (*x*-axis), averaged over the density and group difference parameters, which had negligible effects on runtime. BGGM exhibits minimal runtime across all conditions. NCT runtime increases with the number of variables but is largely unaffected by sample size. In contrast, the runtime for both variants of our method increases with both the number of variables and the sample size.

### 5.3 Discussion of simulation results

Our simulation results demonstrate that all methods recover group differences at rates well above chance across realistic conditions. Across network densities, the proposed methods based on the ordinal MRF consistently achieved the highest performance, with the beta-Bernoulli prior yielding slightly higher AUCs than the Bernoulli prior. For the fully connected networks, BGGM performed next best, while the NCT exhibited the lowest performance. In the sparse network condition, NCT slightly outperformed BGGM, although both remained clearly below the proposed methods in overall accuracy.

For applied researchers aiming to test group differences in empirical ordinal data, these results suggest that, when the ordinal MRF provides a reasonable model for the data, the proposed method offers substantial advantages over the alternatives. This holds even in sparse network settings, despite the fact that sparsity is not an explicit modeling assumption of our approach.

The standard error of the AUC estimates ranged from 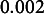 to 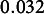 (in conditions with low *n*), with an average of 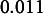 . We consider this level of uncertainty acceptable given the contrasts discussed. For example, the smallest AUC difference we report—between bgms(BB) and bgms(B)—is larger than the average standard error. Moreover, the consistency in relative performance across the majority of conditions suggests that these patterns are not attributable to sampling variability.

As with any simulation study, the generalizability of our findings to unexamined scenarios cannot be guaranteed. However, because our data-generating models are calibrated using empirical data from a large and typical dataset, we expect the conclusions to extend to settings with similar characteristics.

## 6 Discussion

In this article, we have introduced a new Bayesian method for analyzing two-group differences in networks of ordinal variables, addressing the limitations of existing network approaches that focus on Gaussian variables. We extended the OMRF framework to model group-specific networks and developed a novel Markov chain Monte Carlo (MCMC) procedure to estimate model parameters. This procedure enables Bayes factor tests for both parameter equivalence and differences across groups. Three numerical checks confirmed that the proposed methods are correctly implemented in the bgms R package (Marsman et al., 2024) and that they worked as intended. Moreover, the method uniquely supports testing differences in category thresholds, a capability absent in other approaches. Together, these features enable a richer approach to analyzing network differences in binary and ordinal data.

To evaluate performance in practical settings, we conducted a large-scale simulation study comparing our method to the NCT (van Borkulo et al., 2023) and the Bayes factor approach in BGGM (Williams et al., 2020). Overall, the proposed method outperformed existing alternatives in detecting group differences in interaction parameters across most conditions, including both dense and sparse networks. In particular, the proposed Bayesian method showed excellent performance even in small sample sizes, a valuable feature that the alternatives could not achieve. While performance differences diminished with increasing sample size, and NCT was competitive, occasionally matching our method’s performance, in large-sample, low-dimensional settings (e.g., large *n* and small *p*), our approach consistently performed best in the majority of realistic empirical scenarios. These results suggest that the proposed Bayesian method offers robust and accurate inference across a wide range of conditions encountered in psychological research.

A major advantage of the proposed Bayes factor approach over traditional non-Bayesian methods is its ability to quantify evidence both for and against individual effects. This allows for robust Bayesian tests of conditional dependence or independence in one-group MRF models, as well as tests of parameter equivalence or difference across two independent groups. In contrast, commonly used network comparison methods, such as the NCT, are often interpreted as providing evidence for the null (e.g., no group differences), but in fact do not offer a principled way to support the null hypothesis. The ability to quantify support for parameter equality is a key strength of the Bayes factor approach and sets it apart from existing frequentist alternatives.

The inclusion Bayes factor we propose averages over all possible models, providing a robust approach in the presence of model uncertainty. This property likely contributed to the strong performance of our method, particularly in small sample settings. A significant advantage of the inclusion Bayes factor is its principled handling of multiple testing. When combined with a hierarchical prior on the number of differences, such as the beta-Binomial prior, it automatically accounts for model complexity and the multiplicity of tested hypotheses. This leads to a form of multiplicity control that reduces the likelihood of false discoveries without requiring arbitrary threshold adjustments (Scott & Berger, 2010). In our simulations, this mechanism likely explains the slightly better performance of the beta-Bernoulli model compared to the fixed-inclusion Bernoulli model, and we expect similar advantages over single-model Bayes factor approaches, which do not address multiple testing in the same principled way.

There are several modeling limitations that we would like to acknowledge and address in future developments. Ideally, we would have a single inferential framework for network analysis that covers the full range of psychometric variables and designs. Currently, however, the proposed framework can only account for binary and/or ordinal variables. Although these are the most common types of variables in psychological network analysis, we would like to extend the framework, and thus the underlying models, to other types of variables, such as continuous variables. The conditional GGM (Lauritzen & Wermuth, 1989) provides a concrete approach for doing so. Another limitation of the model is that it only considers designs with two independent groups. Extending the underlying idea of an independent samples *t* test to an analysis of variance (ANOVA) design, following the ideas underlying Bayesian ANOVA (Rouder et al., 2012), provides a concrete direction for designs with more than two independent samples. The extension to dependent samples (e.g., a pre- and post-intervention design) requires a new modeling strategy involving random intercepts. All of these limitations provide concrete directions for future research.

There are also limitations and opportunities for improvement in the computational methodology. Numerical Check I showed that the Metropolis algorithms used to update the model parameters were much less efficient than rstan. While the Metropolis approach that we used is flexible and broadly applicable, it can result in slow mixing, leading to high autocorrelation or a low effective sample size in the sampled chains. To address this issue, we are exploring advanced sampling strategies, including gradient-based methods such as the Metropolis-adjusted Langevin algorithm (Besag, 1994; Rossky et al., 1978). A second limitation is our reliance on the pseudolikelihood to avoid computing the intractable normalization constant. While the pseudolikelihood leads to consistent posteriors, it may balance sensitivity and specificity differently than the full likelihood (e.g., Keetelaar et al., 2024; Marsman et al., 2025). We are investigating other approximations to the full likelihood to better capture its information. Despite these computational limitations, our numerical experiments showed that the proposed methodology performs well and outperforms existing alternatives under most conditions.

We have implemented the proposed Bayesian methods in the R package bgms (version 0.1.4.2 Marsman et al., 2024), which can be installed from CRAN (see https://cran.r-project.org/web/packages/bgms/index.html). The computational aspects of the software are mostly written in C++ using the Rcpp package (Eddelbuettel, 2013). In a series of numerical experiments, we have shown that the proposed methodology works and is correctly implemented in the R package. However, the timing results from our simulations revealed that the method can be computationally intensive, particularly when the number of variables or sample size is large. Although the implementation benefits from C++-level optimization, the runtime can still be substantially longer than that of existing alternatives. We are therefore continuously working to improve computational efficiency, both by developing more efficient sampling algorithms and by refining the underlying software implementation. In addition to these efforts, we are currently integrating the independent samples comparison into the easybgm R package (Huth et al., 2024) to streamline the analysis, and the bgms package into the JASP software (Love et al., 2019; Wagenmakers et al., 2018) so that applied researchers without R experience can use the methodology.

## 7 Conclusion

We proposed a new two-group extension of the OMRF that allows researchers to model group differences in both pairwise interactions and category thresholds in networks of binary and ordinal variables. Alongside this model, we introduced a Bayesian methodology for estimation and inference, implemented in the bgms package, that enables principled evaluation of these differences using empirical data. The proposed methods provide researchers with a coherent and flexible framework for assessing group differences in network structures. By offering both a suitable modeling framework for ordinal data and a robust inferential approach, we aim to help place the growing literature on psychological networks on a firmer methodological foundation.

## Figures and Tables

**Figure C1:**
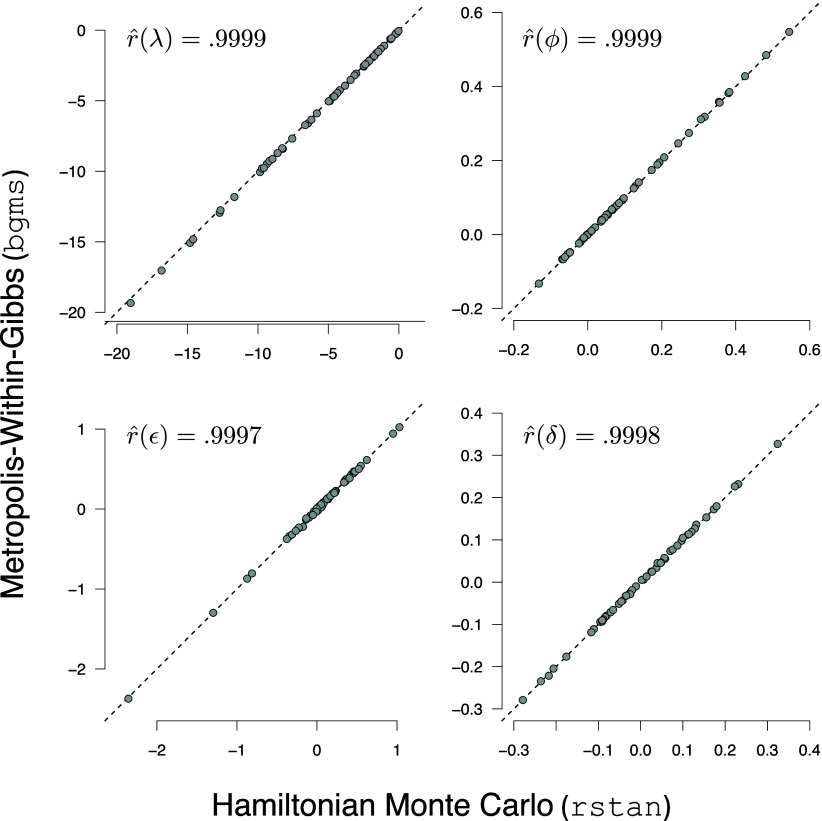
Scatterplots of the posterior means estimated using the Hamiltonian Monte Carlo approach, as implemented in the R package rstan, against the posterior means estimated using the Metropolis-within-Gibbs approach, as implemented in the R package bgms.

**Figure C2:**
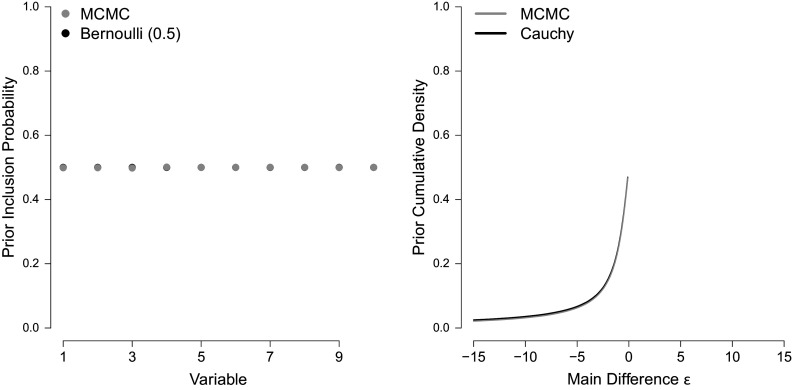
The estimated prior inclusion probability of the threshold differences based on the outlined procedure is shown in the left panel, and the estimated prior density for one of the threshold difference parameters is shown in the right panel.

**Figure C3:**
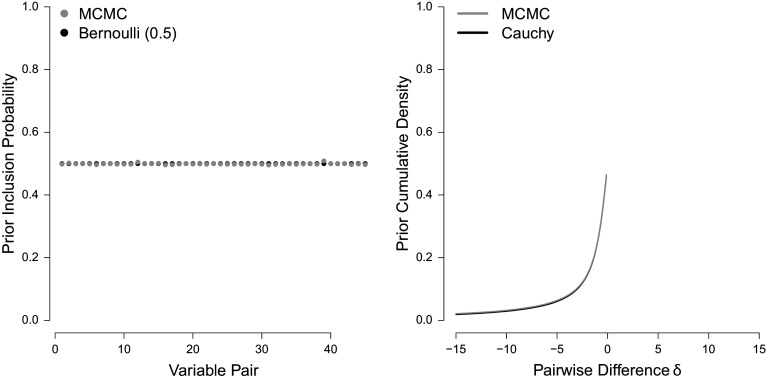
The estimated prior inclusion probability of the pairwise differences based on the outlined procedure is shown in the left panel, and the estimated prior density for one of the pairwise difference parameters is shown in the right panel.

**Figure C4:**
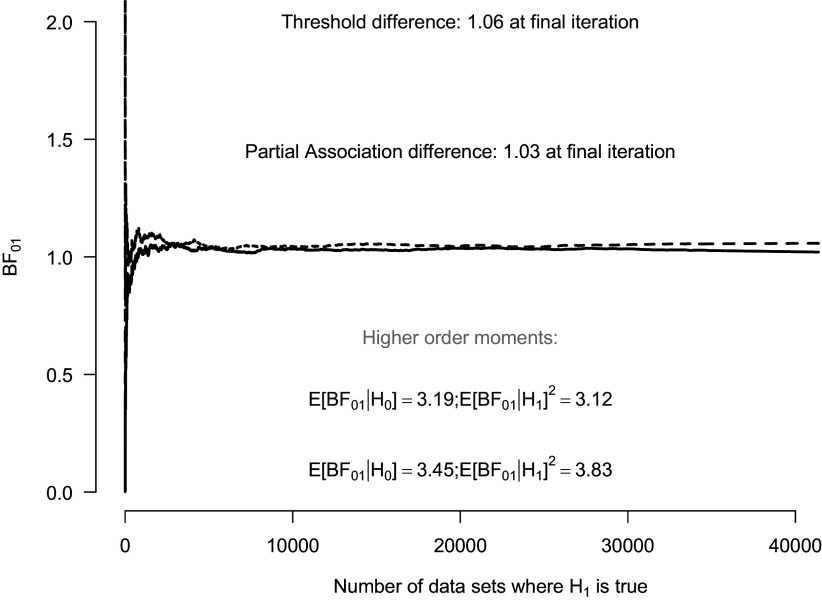
Monte Carlo estimates of the expected Bayes factor 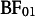 as a function of the number of synthetic data sets generated under 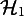 . *Note:* Both the threshold and the pairwise difference Bayes factors tend to one. In addition, the first- and second-order moments are similar.

## Footnotes

1 To simplify the notation, we assume here that all variables have the same number of response categories.

## Supplementary material

The supplementary material for this article can be found at https://doi.org/10.1017/psy.2025.10060.

## Appendix

### A Parameter identification of MRF models for ordinal data

Marsman et al. (2025, Section 2.4) showed that the parameters of the OMRF are identified, that is, that the corresponding probability distribution  in Eq. (1) is an injective function of the parameters. We first give a new proof for the identification of the OMRF, which gives us a strategy to show that the parameters of the two-group OMRF are also identified.

#### A.1 Parameter identification of the ordinal MRF

The OMRF is in the exponential family and has sufficient statistics for each parameter. The sufficient statistics for the category thresholds  are the indicator functions  , for  (i.e., we set  for identification, see below), and the sufficient statistics for the pairwise interactions are the pairwise products  . We collect all these statistics in the vector  , where  . In the same way, we collect the corresponding parameters  and  in the vector  . We can then rewrite the probability distribution characterized by the OMRF as

We show that the elements in  have no linear constraints, that is, that they cannot be expressed as a linear function of each other, which implies the identifiability of the parameter vector  (Lehmann & Casella, 1998).

For each variable, we set the threshold parameter for the first category to a constant for identification (i.e.,  ). If we had not done this, then the sufficient statistics for the category thresholds of a variable *i* would satisfy the linear constraint:

and the parameter vector  would not be identifiable. By setting the threshold parameter of the first category to the constant zero for each variable in the network, the statistic  is not part of  . This implies that the value of the sufficient statistic for the threshold parameter of a category *c* cannot be a linear combination of the sufficient statistics corresponding to the remaining categories (or pairwise interactions) alone. That is, they no longer satisfy linear constraints. Similarly, the statistics corresponding to the pairwise interactions cannot be expressed as a linear combination of the remaining statistics. To see why, consider the statistic  that corresponds to the pairwise interaction parameter  . If it can be expressed as a linear combination of the remaining statistics, then this relation must hold for all possible realizations of the vector  . Now let’s assume that all other variables are zero, but that  . In this case, the only non-zero statistics we have are  ,  , and  for some integers  , and there is clearly no linear combination of  and  that can reproduce the statistic  . In the same way, one can show that there is no linear combination that can reproduce the statistic  if the remaining variables are all one, or some are one and some are zero. This shows that the pairwise interactions also have no linear constraint, and the parameter vector  is identifiable.

#### A.2 Parameter identification of the two-group ordinal MRF

Since the parameters of the two-group MRF are a one-to-one function of the parameters of two independent MRFs, and since we have established that the parameters of the independent MRFs are identifiable, we know that the parameters of the two-group MRF must also be identifiable. To verify this, we follow the same strategy as before and focus on the comparison of the OMRF in two groups.

The two-group OMRF is in the exponential family and has sufficient statistics for each parameter. The sufficient statistics for the category threshold parameters  are  , the sufficient statistics for the category threshold differences  are , the sufficient statistics for the pairwise interactions  are the sum of the pairwise products  , and for the pairwise differences  the differences of the pairwise products . We again collect all these statistics in the vector  , where  , and collect the corresponding parameters  ,  ,  , and  in the vector  . Proof of the identifiability of the parameter vector  follows the same steps and arguments as above.

For reasons stated above, we set the category threshold parameter and category threshold difference parameter for the first category to a constant for identification (i.e.,  and  ) for each variable. Then, it is clear from the sufficient statistics defined above, that  , for  and every  . That is, we cannot express the sufficient statistics for the category thresholds and their differences as a linear combination of each other or of the remaining statistics.

Similarly, the statistics corresponding to the pairwise interactions and their differences cannot be expressed as a linear combination of the remaining statistics. To see why, consider the statistic  that corresponds to the pairwise interaction parameter  . If it can be expressed as a linear combination of the remaining statistics, then this relation must hold for all possible realizations of the vectors  and  . Now, let’s assume that all other variables are zero, but that  . In this case, the non-zero statistics we have are  , ,  ,  , ,  for some integers  . There is no linear combination of these five statistics that can reproduce the statistic  for every possible realization of  and  . Let’s examine a single example to verify that this statement is true. Let  , which implies that either  and at least one of  and  is zero (scenario 1), or at least one of  and  is zero and  (scenario 2). In scenario 1, we have that ,  is one or two,  is one or two,  is zero or minus a half, and  is zero or minus a half. In scenario 2, we have that ,  is one or two,  is one or two,  is zero or a half, and  is zero or a half. From this, we see that in neither scenario can we uniquely express the statistic  as a linear function of the other statistics.

Since none of the statistics discussed so far could be expressed as a linear combination involving the pairwise difference statistic, the converse must also hold. That is, the pairwise difference statistic cannot be expressed as a linear function of any of the statistics discussed so far. The argument for the sum of the pairwise products can also be used for the difference of the pairwise products to argue that none of the pairwise difference statistics can be expressed as a linear combination of the remaining differences in the pairwise products. Thus, the parameter vector  is identifiable.

### B Full conditional simulation steps for the Gibbs sampler

#### B.1 Sampling category thresholds via independence Metropolis

The full conditional pseudoposterior of the category threshold parameters are of the form

for  , and  . Here, we have used the following shorthand notations  ,  , and

The full conditional resembles a generalized beta-prime distribution

Maris et al. (2015) suggested using the beta-prime as the proposal in a Metropolis algorithm, and using the properties of the target distribution to set the proposal parameters. We have successfully used this idea before to simulate the threshold parameters of the IGM and OMRF (e.g., Marsman et al. 2022, 2025).

The log of the target distribution is concave and has linear tails:

and the same holds for the proposal distribution:

We match the tails of the proposal distribution to that of the target distribution by setting  and  . The last free parameter,  , is used to ensure that the proposal closely matches the target distribution at the current state of the Markov chain. Specifically,  is used to make the derivatives of the logarithms of the proposal and target distributions equal. If  is the current state of  in the Markov chain, then we equate the two derivatives and solve for *d*, which yields

Now that we have the value for  , we can sample a proposal from the generalized beta-prime distribution in the following way: we sample *W* from a Beta  distribution, and set the proposed value  equal to  . Here,  is a sample from the generalized beta-prime distribution. We accept the proposed value with probability

and retain the current value  otherwise.

#### B.2 Sampling threshold differences via adaptive Metropolis

The full conditional pseudoposterior distribution of the threshold difference parameters are of the form

where  is the scale of the Cauchy prior. We will sample from these full conditional pseudoposterior distributions using adaptive Metropolis (Atchadé & Rosenthal, 2005; Griffin & Steel, 2022).

The adaptive Metropolis algorithm is implemented as follows. We propose a new value  from a normal density centered at the current state  . The variance of this proposal density is  . We accept the proposed value with probability

and retain the current value otherwise.

The variance of the proposal distribution is calibrated using a algorithm (Robbins–Monro 1951). Specifically, at initialization *t* of the Gibbs sampler, we set the logarithm of the proposal variance  equal to

where  was the acceptance probability in the current Metropolis step. This approach adjusts the proposal variance so that the Metropolis algorithm’s target acceptance probability is  , which is generally considered optimal for a Metropolis–Hastings random walk.

#### B.3 Sampling pairwise interactions via adaptive Metropolis

The full conditional pseudoposterior distribution of the pairwise interactions are of the form

where  is the scale of the Cauchy prior.

The adaptive Metropolis algorithm to simulate from these pseudoposterior distributions is implemented as follows. We propose a new value  from a normal density centered at the current state  . The variance of this proposal density is  . We accept the proposed value with probability

and update the proposal variance  using the algorithm (Robbins–Monro 1951) as outlined in the previous section of this Appendix (i.e., B.2).

#### B.4 Sampling pairwise differences via adaptive Metropolis

The full conditional pseudoposterior distribution of the pairwise differences are of the form

where  is the scale of the Cauchy prior.

The adaptive Metropolis algorithm to simulate from these pseudoposterior distributions is implemented as follows. We propose a new value  from a normal density centered at the current state  . The variance of this proposal density is  . We accept the proposed value with probability

and update the proposal variance  using the algorithm (Robbins–Monro 1951) as outlined in a previous section of this Appendix (i.e., B.2).

#### B.5 Joint sampling of threshold differences and indicators

We use a Metropolis step and propose a new value for the difference indicator  and the difference parameters  based on their current states (i.e.,  and  ). We factor the proposal distribution as

We will also use the MoMS formulation for the two proposal distributions. First, we consider the proposal distribution for the edge indicator,

which suggests changing the edge, i.e., it sets  to make the between model move. Next, we define the conditional proposal distribution for the threshold difference effect,

Here, the proposal value  is equal to  if  , otherwise, it is drawn from a proposal density  . We use the adaptive proposals from the adaptive Metropolis (cf. Appendix B.2).

Let  and  denote the current states of the threshold difference indicator and the threshold difference parameter vector for the variable *i*, and let  and  denote their proposed states. We accept the proposed states with probability  , where

where  is the current state of the matrix of threshold differences and  its proposed state with elements in row *i* set equal to the proposed value  . If  , we propose  and  , for  , and accept the proposed values with probability

Conversely, if  , we propose  and  . We accept  and  with probability

#### B.6 Joint sampling of pairwise differences and indicators

Let  and  denote the current states of the pairwise difference indicator and the pairwise difference parameter for the variables *i* and *j*, and let  and  denote their proposed states. We use the adaptive proposals from the adaptive Metropolis (cf. Appendix B.4). We accept the proposed states with probability  , where

where  is the current state of the matrix of pairwise differences and  is its proposed state, with elements in row *i* column *j* and row *j* column *i* set equal to the proposed value  . If  , we propose  and  , and accept the proposed values with probability

Conversely, if  , we propose  and  . We accept  and  with probability

### C Numerical checks

The R scripts used for the numerical checks and the rstan implementation used in the first numerical check can be found at https://osf.io/txhbp/overview?view_only=0a2ed24ecc4448fbba104faa28e6f8c7.

#### C.1 Numerical Check I: Estimating the pseudoposterior with bgms and rstan

Our approach to Bayesian hypothesis testing about parameter differences between the two groups relies on the numerical procedures for Bayesian parameter estimation. Our software implementation consists of more than  lines of C++ code, which calls for a careful evaluation of whether the software works correctly. To verify that these numerical procedures are implemented correctly, we compare their estimates with the estimates from an alternative software implementation. Here, we consider the popular Stan language (Gelman et al., 2015) using the R package rstan (Stan Development Team, 2024a), which is a general-purpose probabilistic software package for Bayesian estimation in which the user can code their preferred model and prior distributions, and the software compiles a Hamiltonian Monte Carlo (HMC) procedure to simulate from the corresponding posterior distribution (Hoffman & Gelman, 2014; Neal, 1996).

Our rstan implementation for simulating from the pseudoposterior distribution of the parameters of the two-group OMRF, which must also use the pseudolikelihood approximation, is new and can be used to as an alternative to bgms to analyze the two-group OMRF. However, the caveat of this software, which is the limitation of the HMC method implemented in Stan, is that it cannot handle discrete parameters, such as the indicator variables used in the Bayesian variable selection methods that we will use later to construct Bayes factor hypothesis tests about parameter differences between the two groups. However, the Savage-Dickey Bayes factor can be computed from the Stan output, which we will use as an alternative to the inclusion Bayes factor, which we can compute from the output of a fully Bayesian specification, that is, one that also takes the structure of the network into account.

To compare estimates from both implementations, we use the empirical dataset reported by Adamkovic et al. (2022), in which the authors assessed life satisfaction, posttraumatic growth, coping strategies, and resilience in 696 cancer survivors. We consider an analysis in which they contrasted the network structure of (ordinal) responses to five items of the Satisfaction with Life Scale and six items of the Brief Resilience Scale between men and women. Their data are openly available at an online repository at https://osf.io/gxrje. We reanalyzed their data using the two-sample OMRF with the HMC procedure as implemented in the R package rstan and the MwG procedure as implemented in the R package bgms. The HMC procedure was run using the rstan defaults of four separate Markov chains, each running for  iterations with  burnin iterations, and the MwG procedure was run using a chain running for  iterations with  burnin iterations. Scatter plots of the posterior means estimated by the two methods are displayed in Figure 5, along with the correlation of their estimates, showing that the results are nearly identical for the two methods. Additional comparisons between the bgms and rstan results are shown in Appendix C.4.

#### C.2 Numerical Check II: Prior recovery

In a second numerical check, we verify that the Bayesian variable selection method works and is implemented correctly. In the absence of data, we know exactly what the target distribution of the MCMC procedure is, so a simple numerical check is to see if the procedures can recover the prior. To this end, we consider two checks. First, we check whether the MCMC procedure can recover the prior inclusion probabilities and the prior densities of the threshold differences in the two-group model. Second, we check whether the MCMC procedure can recover the prior inclusion probabilities and the prior densities of the pairwise differences in the two-group model. Since the procedure for estimating and selecting the pairwise interactions in the one-group model works in exactly the same way, we do not perform this additional check here.

For the numerical check of the threshold difference estimation and selection procedure, we consider a network with ten variables and three response categories (i.e.,  threshold difference parameters per variable). This results in a total of twenty threshold difference parameters and ten difference indicators to be sampled. We ran the MCMC procedure for one million iterations. The estimated prior inclusion probabilities for the ten difference indicators and the cumulative density of the first threshold difference parameter are shown in Figure 6, and show that the method was able to recover both well. There is a slight discrepancy between the tails of the Cauchy prior and the recovered density using a normal proposal. This appears to be unique to the tails of the Cauchy distribution, as additional checks with other types of distributions did not reveal any differences.

For the numerical check of the pairwise difference estimation and selection procedure, we consider a network with ten variables. This results in a total of 45 pairwise difference parameters and difference indicators to be sampled. We ran the MCMC procedure for one million iterations. The estimated prior inclusion probabilities for the 45 difference indicators and the cumulative density of the first pairwise difference parameter are shown in Figure 7 and show that the method was able to recover both well. Again, there is a slight discrepancy between the tails of the Cauchy prior and the recovered density using a normal proposal.

#### C.3 Numerical Check III: A good check on the difference Bayes factors

Our first numerical check verified that the MCMC procedure was implemented correctly by showing that the estimates from bgms were consistent with estimates from an implementation of rstan, and our second check showed that the variable selection methodology was able to recover the prior distributions for the difference indicators and the difference parameters. Next, we want to verify that the MCMC procedure for Bayesian hypothesis testing is implemented correctly. That is, that the inclusion Bayes factor for testing for the presence of differences in MRF parameters between two independent groups is correctly computed. To implement this check, we cannot compare the bgms estimates with the rstan estimates because rstan cannot model the inclusion indicators. Therefore, we have to take a different approach and perform a numerical check proposed by Sekulovski et al. (2024) based on two theorems about the properties of Bayes factors attributed to Alan Turing and Jack Good (e.g., Good, 1984).

The first theorem states that the expected Bayes factor in favor of a false hypothesis is equal to one, that is,  , while the second theorem generalizes this result to higher order moments. Sekulovski et al. (2024) have previously used this method to verify the computational accuracy of the inclusion Bayes factor for testing for the presence of edges in a single group, as proposed in Marsman et al. (2025). We extend this approach to evaluate the computation of Bayes factors for testing differences in category thresholds and pairwise interaction parameters between two independent groups using the difference selection approach.

Following the procedure outlined by Sekulovski et al. (2024), we simulate  data sets with  binary variables for two groups consisting of  cases each, both under  and  . Under  , we simulate the data after setting all overall category threshold parameters  to  , all interaction parameters  to  , all threshold differences  and pairwise differences  to zero, but sampling one threshold difference  and one pairwise difference  from a Cauchy(0,1) distribution. Under  we simulate the data by also setting  and  to zero. We run the MCMC procedure on each of the simulated data sets with  iterations. Of the  analyses,  were successful, and for each of them we compute the inclusion Bayes factor for the threshold differences for variable 1 and for the pairwise difference for variables 4 and 5.

For data sets simulated under  , we compute the cumulative first and second moments of the Bayes factors in favor of  about the origin (i.e.,  and  ) for both the threshold and partial association difference parameters. We also compute the first moment of the Bayes factor in favor of  using the data simulated under  . The results are shown in Figure 8.

Figure 8 shows that both the Bayes factor for the category threshold difference and the Bayes factor for the pairwise difference converge to one. In addition, the mean Bayes factor in favor of the true hypothesis for the data simulated under  is approximately equal to the second moment of the Bayes factor in favor of the false hypothesis for the data simulated under  . According to Sekulovski et al. (2024), this result provides strong evidence for the correct calculation of Bayes factors.

#### C.4 Numerical Check I, continued: Parameter estimates and convergence diagnosis

The first numerical check verified that the MCMC procedure was correctly implemented in bgms, since the posterior means obtained from bgms and rstan were indistinguishable. Here we examine the quality of the MCMC output of the two approaches.

We generated a data set using the same setup we used for Numerical Check III under  . We ran the rstan software with its default settings: Four independent chains of  iterations each, discarding the first  iterations as a warm-up. Similarly, we ran four independent chains of  iterations each for bgms, discarding the first  iterations as a warm-up. This is the default number of iterations for bgms, although unlike rstan, the bgms software runs a single chain by default.

The posterior means and standard deviations for the estimates from the two procedures are listed in Table C1. Again, these are very similar between the two methods.

To assess the convergence of the Markov chains, we compute the Gelman-Rubin statistic (  , Gelman & Rubin, 1992), which compares the within-chain variance to the between-chain variance.  values close to one indicate convergence, but there is some variability in what is considered close to one, with cutoff values ranging from 1.1, 1.05, to 1.01 (e.g. Gelman & Rubin, 1992; Johnson et al., 2022; Vehtari et al., 2021). For our example, the  statistic is equal to or less than 1.01 for all parameters except one, which is equal to 1.02. This indicates satisfactory convergence for the two liberal cutoffs, but not for the most stringent, suggesting that a few more samples are needed.

We also computed the effective sample size (  ), which estimates how many independent samples from the posterior would carry the same information as the dependent samples produced by the two methods. The methods show a striking difference in effective sample sizes (  ), with the rstan samples consistently producing larger  values than the bgms samples, even though the former ran for far fewer iterations than the latter. The Hamiltonian Monte Carlo (HMC) procedure in rstan produces much less autocorrelation between successive states of the Markov chain than the adaptive Metropolis–Hastings within Gibbs procedure in bgms. Note that the  values for the Stan model for some of the parameters exceed the total number of iterations of the sampler (excluding the warm-up). For some estimation problems in Stan, HMC can produce such anticorrelated draws, which can cause the effective sample size to exceed the number of iterations (Stan Development Team, 2024b). This suggests that while the Markov chain converges quickly, it requires many samples to efficiently explore the posterior distribution, e.g., to estimate the tails of the posterior distribution. For this reason, we usually suggest running the MCMC procedure for many more iterations (e.g., 2024).

**Table C1:** Posterior means and standard deviations, and convergence diagnostics, from bgms and rstan output

Parameter	Mean		SD					
	bgms	rstan	bgms	rstan	bgms	rstan	bgms	rstan
	0.37	0.37	0.21	0.22	2,185	3,467	1.00	1.00
	0.32	0.32	0.21	0.21	2,023	3,614	1.00	1.00
	0.38	0.37	0.21	0.21	2,043	3,507	1.01	1.00
	0.72	0.72	0.21	0.22	2,241	3,870	1.01	1.00
	0.23	0.23	0.21	0.21	2,147	3,949	1.00	1.00
	0.43	0.42	0.38	0.38	1,305	3,645	1.01	1.00
	0.17	0.17	0.37	0.37	1,331	4,063	1.00	1.00
	0.56	0.55	0.39	0.38	1,143	3,980	1.00	1.00
	0.34	0.35	0.37	0.39	1,275	3,945	1.00	1.00
	0.71	0.71	0.41	0.40	1,042	4,159	1.00	1.00
	0.35	0.36	0.16	0.16	1,684	4,894	1.01	1.00
	0.59	0.59	0.15	0.16	1,854	4,587	1.02	1.00
	0.85	0.85	0.15	0.15	1,866	4,628	1.00	1.00
	0.35	0.35	0.16	0.16	1,693	4,463	1.00	1.00
	0.58	0.58	0.16	0.15	1,925	4,919	1.01	1.00
	0.67	0.66	0.15	0.16	1,920	4,492	1.00	1.00
	0.17	0.17	0.15	0.16	1,870	4,778	1.01	1.00
	0.26	0.27	0.15	0.16	1,854	5,425	1.00	1.00
	0.45	0.45	0.15	0.15	1,833	4,747	1.01	1.00
	0.54	0.54	0.15	0.16	1,975	4,671	1.01	1.00
	0.12	0.12	0.29	0.29	1,743	4,412	1.00	1.00
	0.07	0.07	0.28	0.29	1,772	5,335	1.01	1.00
	0.18	0.18	0.28	0.29	1,934	4,712	1.00	1.00
	0.24	0.25	0.29	0.30	1,701	4,870	1.00	1.00
	0.24	0.23	0.28	0.28	1,813	5,514	1.00	1.00
	0.12	0.13	0.28	0.29	1,905	5,049	1.00	1.00
	0.38	0.39	0.29	0.28	1,552	4,417	1.00	1.00
	0.16	0.15	0.29	0.29	1,744	4,655	1.00	1.00
	0.73	0.72	0.29	0.30	1,826	4,671	1.00	1.00
	0.19	0.20	0.29	0.29	1,674	4,543	1.00	1.00

*Note*: Results are rounded to two decimal places.

## References

[r1] Adamkovič, M. , Fedáková, D. , Kentoš, M. , Bozogáňová, M. , Havrillová, D. , Baník, G. , Dedová, M. , & Piterová, I. (2022). Relationships between satisfaction with life, posttraumatic growth, coping strategies, and resilience in cancer survivors: A network analysis approach. Psycho-Oncology, 31, 1913–1921. 10.1002/pon.5948. 35524705 PMC9790334

[r2] Agresti, A. (2010). Analysis of ordinal categorical data. (2^nd^ ed.) John Wiley & Sons, Inc. 10.1002/9780470594001

[r3] Agresti, A. (2018). An introduction to categorical data analysis. (3^rd^ ed.). Wiley.

[r4] Anderson, C. J. , & Vermunt, J. K. (2000). Log-multiplicative association models as latent variable models for nominal and/or ordinal data. Sociological Methodology, 30(1), 81–121. 10.1111/0081-1750.00076

[r5] Atchadé, Y. F. , & Rosenthal, J. S. (2005). On adaptive Markov chain Monte Carlo algorithms. Bernoulli, 11(5), 815–828. 10.3150/bj/1130077595

[r6] Barber, R. F. , & Drton, M. (2015). High dimensional Ising model selection with Bayesian information criteria. Electronic Journal of Statistics, 9(1), 567–607. 10.1214/15-EJS1012

[r7] Besag, J. (1975). Statistical analysis of non-lattice data. Journal of the Royal Statistical Society Series D (The Statistician), 24(3), 179–195. 10.2307/2987782

[r8] Besag, J. (1994). Discussion of the paper “representations of knowledge in complex systems” by U. Grenander and M. I. Miller. Journal of the Royal Statistical Society. Series B (Methodological), 56(4), 591–592.

[r9] Billieux, J. , Heeren, A. , Rochat, L. , Maurage, P. , Bayard, S. , Bet, R. , Besche-Richard, C. , Challet-Bouju, G. , Carré, A. , Devos, G. , & Flayelle, M. (2021). Positive and negative urgency as a single coherent construct: Evidence from a large-scale network analysis in clinical and non-clinical samples. Journal of Personality, 89(6), 1252–1262. 34114654 10.1111/jopy.12655 PMC9292904

[r10] Borsboom, D. , Deserno, M. K. , Rhemtulla, M. , Epskamp, S. , Fried, E. I. , McNally, R. J. , & Waldorp, L. J. (2021). Network analysis of multivariate data in psychological science. Nature Reviews Methods Primers, 1(1), 58. 10.1038/s43586-021-00055-w

[r11] Consonni, G. , Fouskakis, D. , Liseo, B. , & Ntzoufras, I. (2018). Prior distributions for objective Bayesian analysis. Bayesian Analysis, 13(2), 627–679. 10.1214/18-BA1103

[r12] Csiszár, I. , & Talata, Z. (2006). Consistent estimation of the basic neighborhood of Markov random fields. The Annals of Statistics, 34(1), 123–145. 10.1214/009053605000000912

[r13] Cyders, M. A. , Littlefield, A. K. , Coffey, S. , & Karyadi, K. A. (2014). Examination of a short english version of the UPPS-P impulsive behavior scale. Addictive Behaviors, 39(9), 1372–1376. 24636739 10.1016/j.addbeh.2014.02.013 PMC4055534

[r14] Danaher, P. , Wang, P. , & Witten, D. M. (2014). The joint graphical lasso for inverse covariance estimation across multiple classes. Journal of the Royal Statistical Society: Series B (Statistical Methodology, 76(2), 373–397. 10.1111/rssb.12033 24817823 PMC4012833

[r15] Dellaportas, P. , Forster, J. J. , & Ntzoufras, I. (2002). On Bayesian model and variable selection using MCMC. Statistics and Computing, 12, 27–36. 10.1023/A:1013164120801199

[r16] Dempster, A. (1972). Covariance selection. Biometrics, 28, 157–175.

[r17] Dickey, J. M. (1971). The weighted likelihood ratio, linear hypotheses on normal location parameters. The Annals of Mathematical Statistics, 42(1), 204–223. 10.1214/aoms/1177693507

[r18] Eddelbuettel, D. (2013). Seamless R and C++ integration with Rcpp. Springer. 10.1007/978-1-4614-6868-4

[r19] Epskamp, S. , Isvoranu, A. , & Cheung, M. (2022). Meta-analytic Gaussian network aggregation. Psychometrika, 87, 12–46. 10.1007/s11336-021-09764-3 34264449 PMC9021114

[r20] Fawcett, T. (2006). An introduction to ROC analysis. Pattern Recognition Letters, 27(8), 861–874. 10.1016/j.patrec.2005.10.010

[r21] Gelman, A. , Lee, D. , & Guo, J. (2015). Stan: A probabilistic programming language for Bayesian inference and optimization. Journal of Educational and Behavioral Statistics, 40, 530–543. 10.3102/1076998615606113

[r22] Gelman, A. , & Rubin, D. B. (1992). Inference from iterative simulation using multiple sequences. Statistical Science, 7(4), 457–472. 10.1214/ss/1177011136

[r23] Geman, S. , & Geman, D. (1984). Stochastic relaxation, Gibbs distributions, and the Bayesian restoration of images. IEEE Transactions on Pattern Analysis and Machine Intelligence, 6(6), 721–741. 10.1109/TPAMI.1984.4767596 22499653

[r24] George, E. I. , & McCulloch, R. E. (1993). Variable selection via Gibbs sampling. Journal of the American Statistical Association, 88(423), 881–889. 10.1080/01621459.1993.10476353

[r25] Good, I. J. (1984). C205. Monotonic properties of the moments of a Bayes factor and the relationship to measures of divergence. Journal of Statistical Computation and Simulation, 19(4), 320–325. 10.1080/00949658408810747

[r26] Gottardo, R. , & Raftery, A. E. (2008). Markov chain Monte Carlo with mixtures of mutually singular distributions. Journal of Computational and Graphical Statistics, 17(4), 949–975. 10.1198/106186008X386102

[r27] Green, P. J. (1995). Reversible jump Markov chain Monte Carlo computation and Bayesian model determination. Biometrika, 82(4), 711–732. 10.1093/biomet/82.4.711

[r28] Griffin, J. E. , & Steel, M. F. J. (2022). Adaptive computational methods for Bayesian variable selection. In M. G. Tadesse , & M. Vanucci (Eds.), Handbook of Bayesian variable selection. CRC Press.

[r29] Haslbeck, J. M. B. (2022). Estimating group differences in network models using moderation analysis. Behavior Research Methods, 54, 522–540. 10.3758/s13428-021-01637-y 34291432 PMC8863727

[r30] Haslbeck, J. M. B. , & Waldorp, L. J. (2020). Mgm: Estimating time-varying mixed graphical models in high-dimensional data. Journal of Statistical Software, 93(8), 1–46. 10.18637/jss.v093.i08

[r31] Hinne, M. , Gronau, Q. F. , van den Bergh, D. , & Wagenmakers, E.-J. (2020). A conceptual introduction to Bayesian model averaging. Advances in Methods and Practices in Psychological Science, 3(2), 200–215. 10.1177/251524591989865

[r32] Hoeting, J. , Madigan, D. , Raftery, A. , & Volinsky, C. (1999). Bayesian model averaging: A tutorial. Statistical Science, 14(4), 382–401.

[r33] Hoffman, M. D. , & Gelman, A. (2014). The No-U-Turn sampler: Adaptively setting path lengths in Hamiltonian Monte Carlo. Journal of Machine Learning Research, 15, 1593–1623.

[r34] Huth, K. B. S. , de Ron, J. , Goudriaan, A. E. , Luigjes, K. , Mohammadi, R. , van Holst, R. J. , & Marsman, M. (2023). Bayesian analysis of cross-sectional networks: A tutorial in R and JASP. Advances in Methods and Practices in Psychological Science., 6(4), 1–18. 10.1177/25152459231193334

[r35] Huth, K. B. S. , Keetelaar, S. , Sekulovski, N. , van den Bergh, D. , & Marsman, M. (2024). Simplifying Bayesian analysis of graphical models for the social sciences with easybgm: A user-friendly R-package. Advances in Psychology, e66366. 10.56296/aip00010

[r36] Ising, E. (1925). Beitrag zur theorie des ferromagnetismus. Zeitschrift für Physik, 31(1), 253–258. 10.1007/BF02980577

[r37] Jamison, L. , Christensen, A. P. , & Golino, H. F. (2024). Metric invariance in exploratory graph analysis via permutation testing. Methodology, 20(2), 144–186. 10.5964/meth.12877

[r38] Jeffreys, H. (1961). Theory of probability. (3^rd^ ed.). Oxford University Press.

[r39] Johnson, A. A. , Ott, M. Q. , & Dogucu, M. (2022). Bayes rules! An introduction to applied Bayesian modeling. CRC Press.

[r40] Kass, R. E. , & Raftery, A. E. (1995). Bayes factors. Journal of the American Statistical Association, 90(430), 773–795. 10.2307/2291091

[r41] Keetelaar, S. , Sekulovski, N. , Borsboom, D. , & Marsman, M. (2024). Comparing maximum likelihood and pseudo-maximum likelihood estimators for the Ising model. Advances in Psychology, 2, e25745. 10.56296/aip00013

[r42] Kindermann, R. , & Snell, J. L. (1980). Markov random fields and their applications (Vol. 1). American Mathematical Society.

[r43] Kuo, L. , & Mallick, B. (1998). Variable selection for regression models. *Sankhyā*: The Indian Journal of Statistics, Series B, 60(1), 65–81.

[r44] Lauritzen, S. , & Wermuth, N. (1989). Graphical models for assocations between variables, some of which are qualitative and some quantitative. The Annals of Statistics, 17(1), 31–57. 10.1214/aos/1176347003

[r45] Lehmann, E. L. , & Casella, G. (1998). Theory of point estimation. (2^nd^ ed.). Springer-Verlag.

[r46] Love, J. , Selker, R. , Marsman, M. , Jamil, T. , Dropmann, D. , Verhagen, A. J. , & Wagenmakers, E.-J. (2019). JASP – Graphical statistical software for common statistical designs. Journal of Statistical Software, 88(2), 1–17. 10.18637/jss.v088.i02

[r47] Maris, G. K. J. , Bechger, T. M. , & San Martin, E. (2015). A Gibbs sampler for the (extended) marginal Rasch model. Psychometrika, 80(4), 859–879. 10.1007/s11336-015-9479-4 26493183 PMC4644215

[r48] Marsman, M. , Arena, G. , Huth, K. B. S. , Sekulovski, N. , & van den Bergh, D. (2024). Bgms: Bayesian analysis of networks of binary and/or ordinal variables [Computer software manual]. (R package version 0.1.4.3). https://cran.r-project.org/package=bgms

[r49] Marsman, M. , Huth, K. B. S. , Waldorp, L. J. , & Ntzoufras, I. (2022). Objective Bayesian edge screening and structure selection for Ising networks. Psychometrika, 87(1), 47–82. 10.1007/s11336-022-09848-8 35192102 PMC9021150

[r50] Marsman, M. , & Rhemtulla, M. (2022). Guest editors’ introduction to the special issue “network psychometrics in action”: Methodological innovations inspired by empirical problems. Psychometrika, 87(1), 1–11. 10.1007/s11336-022-09861-x PMC9021145 35397084

[r51] Marsman, M. , van den Bergh, D. , & Haslbeck, J. M. B. (2025). Bayesian analysis of the ordinal Markov random field. Psychometrika, 90, 146–182. 10.1017/psy.2024.4

[r52] Miller, J. W. (2021). Asymptotic normality, concentration, and coverage of generalized posteriors. Journal of Machine Learning Research, 22(168), 1–53.

[r53] Mohammadi, R. , Schoonhoven, M. , Vogels, L. , & Birbil, I. (2023). Large-scale Bayesian structure learning for Gaussian graphical models using marginal pseudo-likelihood. Preprint. https://arxiv.org/abs/2307.00127

[r54] Neal, R. M. (1996). Monte Carlo implementation. In Bayesian learning for neural networks (Vol. 118, pp. 55–98). Springer. 10.1007/978-1-4612-0745-0_3

[r55] Park, J. , & Haran, M. (2018). Bayesian inference in the presence of intractable normalizing constants. Journal of the American Statistical Association, 113(523), 1372–1390. 10.1080/01621459.2018.1448824

[r56] Pauli, F. , Racugno, W. , & Ventura, L. (2011). Bayesian composite marginal likelihoods. Statistica Sinica, 21(1), 149–164.

[r57] Pensar, J. , Nyman, H. , Niiranen, J. , & Corander, J. (2017). Marginal pseudo-likelihood learning of discrete Markov network structures. Bayesian Analysis, 12(4), 1195–1215. 10.1214/16-BA1032

[r58] Ravikumar, P. , Wainwright, M. J. , & Lafferty, J. D. (2010). High-dimensional Ising model selection using 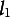 -regularized logistic regression. Annals of Statistics, 38(3), 1287–1319. 10.1214/09-AOS691

[r59] Ribatet, M. , Cooley, D. , & Davison, A. C. (2012). Bayesian inference from composite likelihoods, with an application to spatial extremes. Statistica Sinica, 22(2), 813–845. 10.5705/ss.2009.248

[r60] Robbins, H. , & Monro, S. (1951). A stochastic approximation method. The Annals of Mathematical Statistics, 22(3), 400–407. 10.1214/aoms/1177729586

[r61] Rossky, P. J. , Doll, J. D. , & Friedman, H. L. (1978). Brownian dynamics as smart Monte Carlo simulation. Journal of Chemical Physics, 69(10), 4628–4633. 10.1063/1.436415

[r62] Rouder, J. N. , Morey, R. D. , Speckman, P. L. , & Province, J. M. (2012). Default Bayes factors for ANOVA designs. Journal of Mathematical Psychology, 56(5), 356–374. 10.1016/j.jmp.2012.08.001

[r63] Rouder, J. N. , Speckman, P. L. , Sun, D. , & Morey, R. D. (2009). Bayesian 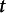 tests for accepting and rejecting the null hypothesis. Psychonomic Bulletin & Review, 16(2), 225–237. 10.3758/PBR.16.2.225 19293088

[r64] Scott, J. G. , & Berger, J. O. (2010). Bayes and empirical-Bayes multiplicity adjustment in the variable-selection problem. Annals of Statistics, 38(5), 2587–2619. 10.1214/10-AOS792

[r65] Sekulovski, N. , Arena, G. , Haslbeck, J. M. B. , Huth, K. B. S. , Friel, N. , & Marsman, M. (2025). A stochastic block prior for clustering in graphical models. https://osf.io/preprints/psyarxiv/29p3m_v1 10.1037/met0000847 42347805

[r66] Sekulovski, N. , Keetelaar, S. , Haslbeck, J. M. B. , & Marsman, M. (2024). Sensitivity analysis of prior distributions in Bayesian graphical modeling: Guiding informed prior choices for conditional independence testing. Advances in Psychology, 2, e92355. 10.56296/aip00016

[r67] Sekulovski, N. , Keetelaar, S. , Huth, K. B. S. , Wagenmakers, E.-J. , van Bork, R. , van den Bergh, D. , & Marsman, M. (2024). Testing conditional independence in psychometric networks: An analysis of three Bayesian methods. Multivariate Behavioral Research, 59, 913–933. 10.1080/00273171.2024.2345915 38733319

[r68] Sekulovski, N. , Marsman, M. , & Wagenmakers, E.-J. (2024). A Good check on the Bayes factor. Behavior Research Methods, 56(8), 8552–8566. 10.3758/s13428-024-02491-4 39231912 PMC11525426

[r69] Stan Development Team. (2024a). *RStan: The R interface to Stan.* (R package version 2.32.6). https://mc-stan.org/

[r70] Stan Development Team. (2024b). Stan reference manual [Computer software manual]. (Version 2.x.) December 4 2024. https://mc-stan.org/docs/reference-manual/

[r71] Suggala, A. S. , Yang, E. , & Ravikumar, P. (2017). Ordinal graphical models: A tale of two approaches. In D. Precup , & Y. W. Teh (Eds.), Proceedings of the 34th international conference on machine learning (Vol. 70, pp. 3260–3269). PMLR.

[r72] Tierney, L. (1994). Markov chains for exploring posterior distributions. The Annals of Statistics, 22(4), 1701–1762. 10.1214/aos/1176325750

[r73] van Borkulo, C. D. , van Bork, R. , Boschloo, L. , Kossakowski, J. J. , Tio, P. , Schoever, R. A. , & Waldorp, L. (2023). Comparing network structures on three aspects: A permutation test. Psychological Methods, 28(6), 1273–1285. 10.1037/met0000476 35404628

[r74] Varin, C. , Reid, N. , & Firth, D. (2011). An overview of composite likelihood methods. Statistica Sinica, 21(1), 5–42.

[r75] Vehtari, A. , Gelman, A. , Simpson, D. , Carpenter, B. , & Bürkner, P. (2021). Rank-normalization, folding, and localization: An improved 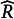 for assessing convergence of MCMC. Bayesian Analysis, 16(2), 667–718. 10.1214/20-BA1221

[r76] Vogels, L. , Mohammadi, R. , Schoonhoven, M. , & Birbil, I. (2024). Bayesian structure learning in undirected gaussian graphical models: Literature review with empirical comparison. Journal of the American Statistical Association, 119, 3164–3182. 10.1080/01621459.2024.2395504.

[r77] Wagenmakers, E.-J. , Love, J. , Marsman, M. , Jamil, T. , Ly, A. , Verhagen, J. , & Morey, R. D. (2018). Bayesian inference for psychology. Part II: Example applications with JASP. Psychonomic Bulletin & Review, 25(1), 58–76. 10.3758/s13423-017-1323-7 28685272 PMC5862926

[r78] Williams, D. R. , & Mulder, J. (2020a). Bayesian hypothesis testing for Gaussian graphical models: Conditional independence and order constraints. Journal of Mathematical Psychology, 99, 102441.

[r79] Williams, D. R. , & Mulder, J. (2020b). BGGM: Bayesian Gaussian graphical models in R. Journal of Open Source Software, 5(51), 2111. 10.21105/joss.02111

[r80] Williams, D. R. , Rast, P. , Pericchi, L. R. , & Mulder, J. (2020). Comparing Gaussian graphical models with the posterior predictive distribution and Bayesian model selection. Psychological Methods, 25(5), 653–672. 10.1037/met0000254 32077709 PMC8572134

